# Design and fabrication of Hong Kong’s first constructional 3D-printed metal pavilion ‘Weaving Love’

**DOI:** 10.1038/s41598-025-17612-y

**Published:** 2025-09-26

**Authors:** Derek Kwok-Leung SO, Billy Chi-Pan WONG, Tony Sai-Kwan SIU, Ning XI, Siu-Lai CHAN, Si-Wei LIU

**Affiliations:** 1 Vibro (H.K.) Ltd., Kowloon, Hong Kong China; 2 Hip Hing Construction Co. Ltd., Kowloon, Hong Kong China; 3 Advanced Technologies Institute, The University of Hong Kong, Pok Fu Lam, Hong Kong China; 4 School of Civil Engineering and Transportation, South China University of Technology, Guangzhou, China; 5 State Key Laboratory of Subtropical Building and Urban Science, South China University of Technology, Guangzhou, China; 6 Department of Civil and Environmental Engineering, The Hong Kong Polytechnic University, Kowloon, Hong Kong China

**Keywords:** Wire arc additive manufacturing (WAAM), Stainless steel structures, Topology optimization, Parametric design, Sustainable construction, Robotic fabrication, Engineering, Civil engineering

## Abstract

‘Weaving Love’ is Hong Kong’s first outdoor pavilion constructed using the constructional 3D metal printing through Wire Arc Additive Manufacturing (WAAM) technology, making a transformative milestone in the application of this emerging technology for large-scale construction in the region. This paper documents the entire process—from the concept and design to fabrication and construction—of the “Weaving Love” pavilion, a constructional 3D-printed metal structure situated at the New Immigration Headquarters of Hong Kong. The project demonstrates the seamless integration of advanced WAAM technology, innovative parametric design, and collaborative efforts among government, industry, and academia. By leveraging these advanced technologies, the project team has created a structure that is not only visually stunning but also environmentally friendly and cost-effective—achievements that would have been unattainable using conventional construction methods. The project achieved significant reductions in construction time, cost, and material waste while pushing the boundaries of architectural design and structural engineering. The structure is one of the largest 3D-printed steel structures in the Hong Kong region, designed in accordance with established codes of practice, just like conventional steel structures. This pioneering project successfully utilized WAAM technology to bring the innovative design of an artistic expression to fruition, incorporating advanced structural analysis methods, optimization techniques, and supplementary physical tests. This paper presents summarized project data and methodological frameworks for implementing WAAM technology in construction applications, thereby contributing to the advancement of WAAM technology in construction industry.

**Supplementary Information:**

The online version contains supplementary material available at 10.1038/s41598-025-17612-y.

## Introduction

**Fig. 1:**
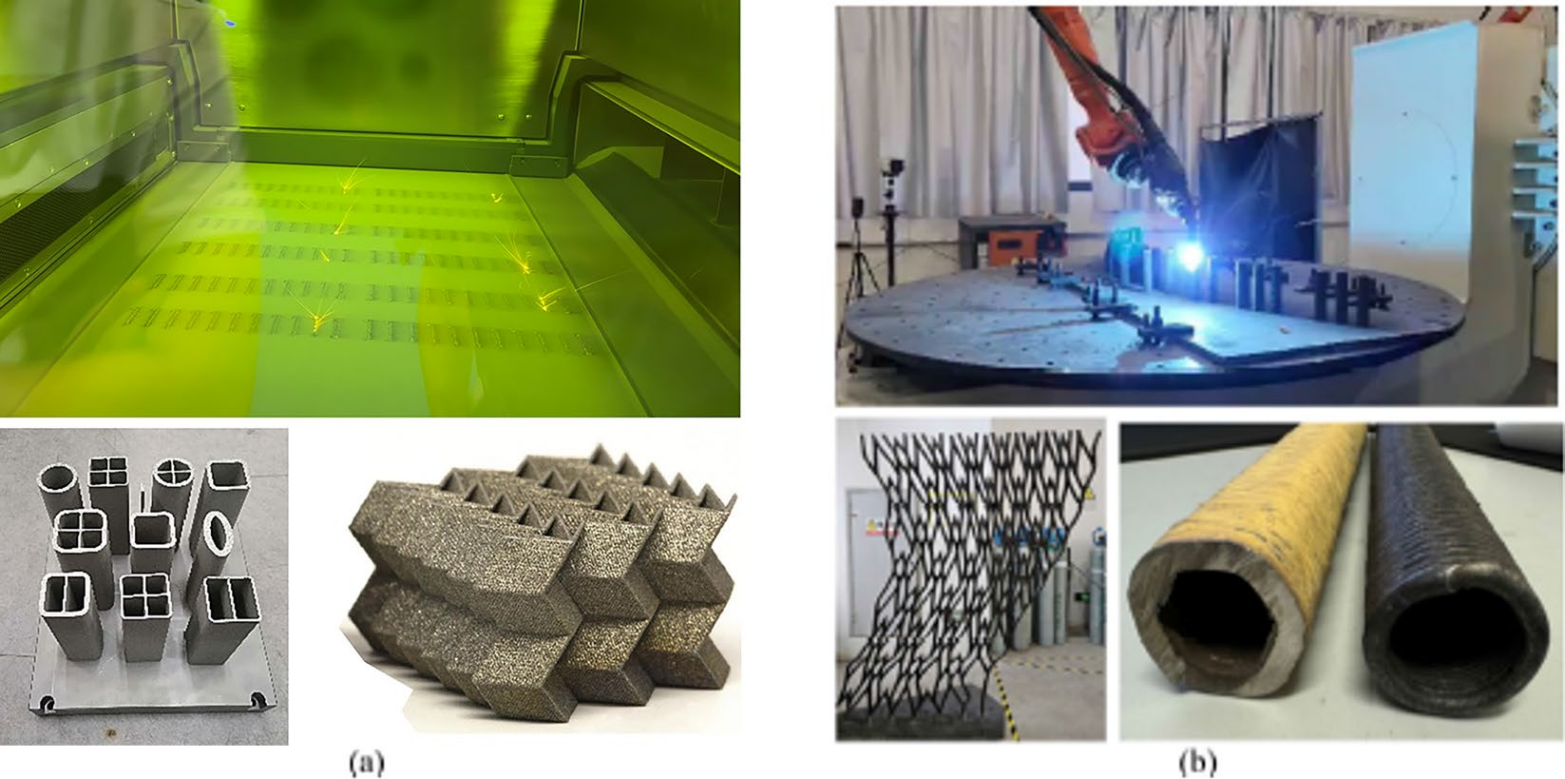
3D metal printing techniques: (**a**) selective laser melting (SLM)^3,4^; (**b**) wire arc additive manufacturing (WAAM).

3D metal printing, also known as metal additive manufacturing, has been progressively integrated into the construction industry over the past decade, offering innovative solutions for building components mostly—particularly joints—its application in large-scale construction remains very limited. The commonly used 3D metal printing techniques are selective laser melting (SLM) and wire arc additive manufacturing (WAAM) (Fig. 1). SLM employs a high-powered laser to selectively fuse metal powders layer by layer, enabling the fabrication of intricate geometries with high precision^1–4^. However, its adoption is often limited by high costs, slow build rates, and size constraints imposed by the fixed dimensions of the powder bed. In contrast, WAAM utilizes an electric arc to melt metal wire, depositing material layer by layer to form the desired component—or even a full-scale structure—depending on the size and capacity of the build platform. This method is particularly advantageous for producing large-scale metal parts as well as the whole structure, offering higher deposition rates and reduced material costs compared to SLM^5,6^. WAAM’s scalability and cost-effectiveness make it especially suitable for applications in construction^7^, where the production of sizable and structurally robust components is essential.

Recent studies have gradually focused on the application of WAAM-fabricated steel structures in the construction industry. Research has various aspects, including material properties^8–10^, member and connection behaviour^11–14^, and structural performance^15^. However, the integration of WAAM into structural design is still challenging. The layer-by-layer deposition process introduces anisotropic material behaviour due to thermal gradients, resulting in variable mechanical properties across different loading orientation^16,17^, which complicates predictions of stiffness and buckling behaviour^18^. Additionally, high deposition rates and thermal effects will result in geometric variations and distortions, such as local thickness changes and global out-of-straightness^19^. Besides, the rapid heating and cooling cycles induce significant residual stresses that are rarely mitigated by heat treatment, unlike in traditional steel^20^. These factors collectively impact the loading capacities of WAAM steel structures. Since WAAM was adopted in this projects, additional physical tests for design parameters were required, including material tests for mechanical properties, 3D scanning for geometric characterisation, member tests for initial imperfections for stability design, etc.

**Fig. 2:**
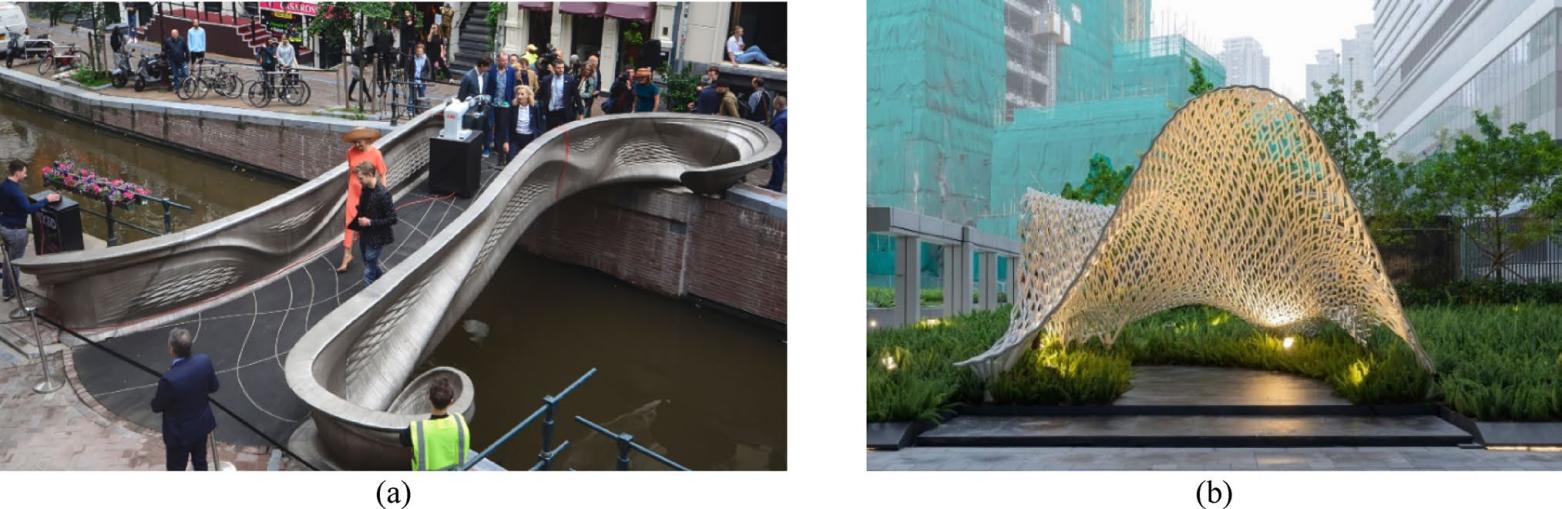
WAAM steel structures: (**a**) MX3D metal 3D-printed footbridge in Amsterdam^21^ (2021–2023); (**b**) Weaving Love pavilion in Hong Kong (2024–Present).

To date, the WAAM technique has been successfully implemented in at least two notable engineering projects (Fig. 2), including the MX3D footbridge in Amsterdam, the “Weaving Love” pavilion in Hong Kong—the focus of this paper. The MX3D footbridge in Amsterdam, unveiled in 2021, is a 12-m-long stainless-steel pedestrian bridge that spans one of Amsterdam’s historic canals. It features a novel, free-form geometry that fully leverages WAAM’s capabilities in fabricating complex structural members. To ensure its stability, full-scale loading test^21^, numerical simulation^22^, and impact hammer testing^23^ have been conducted on this bridge. During its operational stage, a network of sensors to monitor its structural health in real-time, providing valuable data on the performance of 3D-printed metal structures^24^. In Hong Kong, the “Weaving Love” pavilion represents the city’s first large-scale construction project utilizing 3D metal printing technology via WAAM technology. Furthermore, Weaving Love could be the first outdoor sculpture using WAAM technology in Asia, marking a significant milestone in the advancement of 3D metal printing for architectural and artistic applications. This project successfully applied WAAM to realize an innovative design of an artistic expression to fruition, incorporating advanced structural analysis methods, optimization techniques, and supplementary physical tests.

**Fig. 3:**
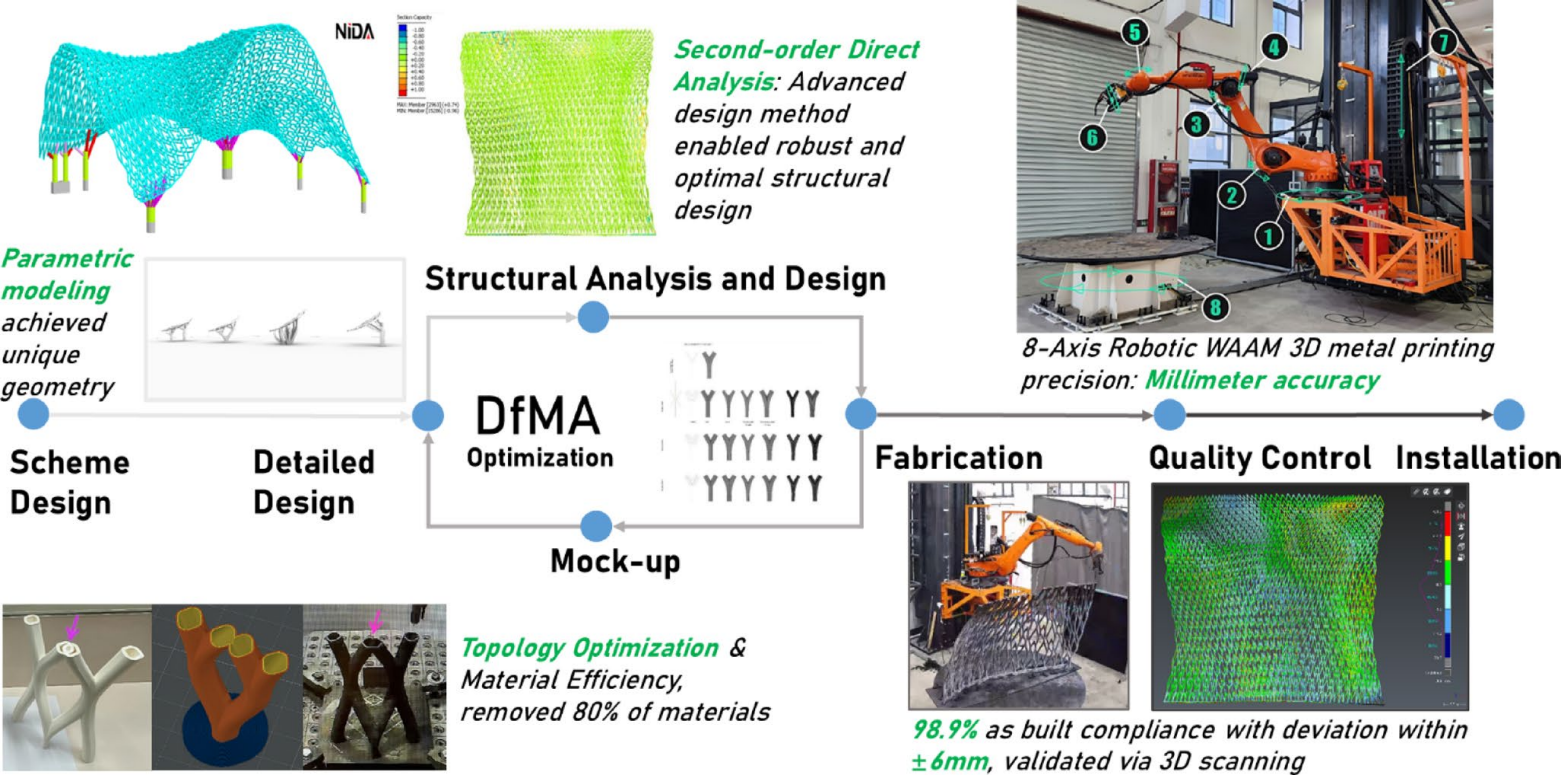
Advanced optimization techniques applied throughout the entire project duration.

Optimization plays a crucial role in the design and construction of WAAM structures, encompassing aspects such as parametric modelling, material usage, and printing strategies^25^ (Fig. 3). Integrating optimization techniques with WAAM can significantly enhance its advantages and unlock its full potential^26^. Advanced optimization approaches have been developed to achieve superior mechanical behaviour with reduced material consumption^27^. By optimizing the design model, material usage, and printing strategies, these approaches improve both the efficiency and structural integrity of WAAM structures. For example, topology optimization enables the creation of lightweight structures that are specifically adopted to the unique mechanical properties of WAAM materials, thereby enhancing material efficiency and overall structural performance^28^. The advanced structural design method, second-order direct analysis^29–31^, was applied using the software platform—NIDA Professional v10^32^ to achieve robust and optimal design without relying on empirical design assumptions such as effective length. Moreover, optimizing the printing strategy can result in more efficient manufacturing paths, reducing production time and material waste^33^. The “Weaving Love” project aims to leverage these optimization techniques to achieve a balance between high mechanical properties and minimal material wastage. By integrating advanced optimization techniques with WAAM, the project demonstrates the potential of this synergy to produce structurally efficient and sustainable designs. The ultimate goal is to showcase how the combination of WAAM and optimization can lead to innovative and practical solutions in modern engineering.

The “Weaving Love” project, represents a pioneering application of WAAM in Hong Kong’s construction industry, addressing these challenges while demonstrating the technique’s practical feasibility. Supported by the Construction Innovation and Technology Fund (CITF) Pioneering Application initiative, this Government-University-Industry (GUI) collaboration framework brought together key stakeholders to materialize the region’s first full-scale 3D Printed metal structure. Located on the upper ground floor of the Immigration Headquarters in Tseung Kwan O, Hong Kong, the pavilion spans approximately 5.0 m in width, 4.8 m in length, and 3.55 m in height, with a total weight of 2.64 tons. Its intricate design, featuring small stainless steel S308L tubes arranged in complex patterns, exemplifies WAAM’s geometric flexibility and underscores its potential to revolutionize structural design. The ‘Weaving Love’ project is documented from its inception to completion, showcasing the technological innovations, design strategies, and collaborative efforts that shaped its success. Through this successful application, valuable insights into the implementation of WAAM in construction and its broader implications for the future of structural engineering in Hong Kong and beyond will be expected.

This paper introduces the construction of the first constructional 3D-printed metal pavilion “Weaving Love” in Hong Kong. The subsequent sections detail the project’s development and conceptualization. Section “Design and concept development” elaborates on the design evolution, encompassing topology optimization and parametric modelling processes. Section “Geometric fine-tuning: deposition rate, surface texture and printability” discusses the printing strategies, including deposition rate, surface texture, and printing path. The structural design and analysis are examined in Section “Structural analysis and design”, followed by the printing fabrication process in Section “Printing fabrication” and the on-site installation in Section “On-site preparation and installation”. Finally, Section “Achievement and future implications” offers an outlook on the future implications of this pioneering project.

## Design and concept development

**Fig. 4:**
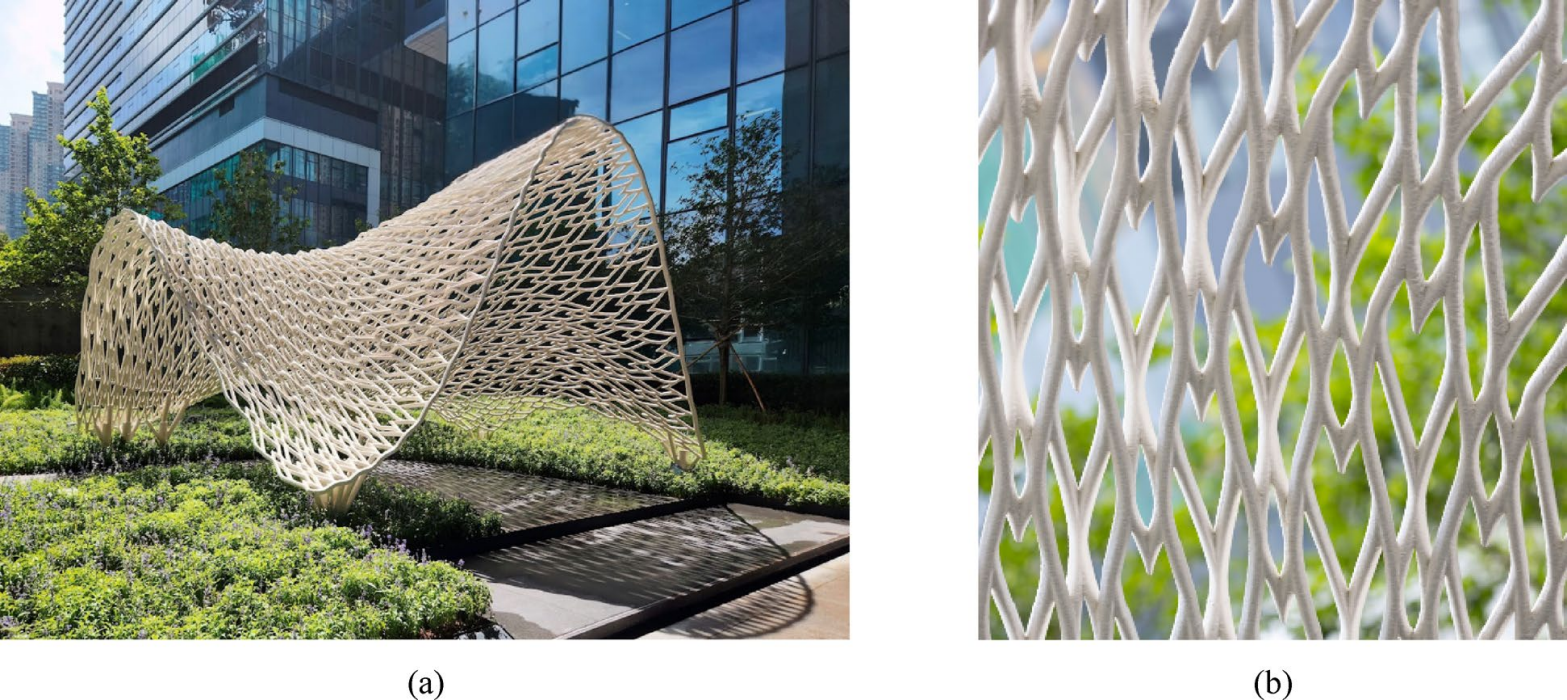
“Weaving Love”, the 3D-printed metal pavilion: (**a**) overall view; (**b**) the natural, non-repeating pattern of the pavilion forms an intricate aesthetics with structural functionality.

**Fig. 5:**
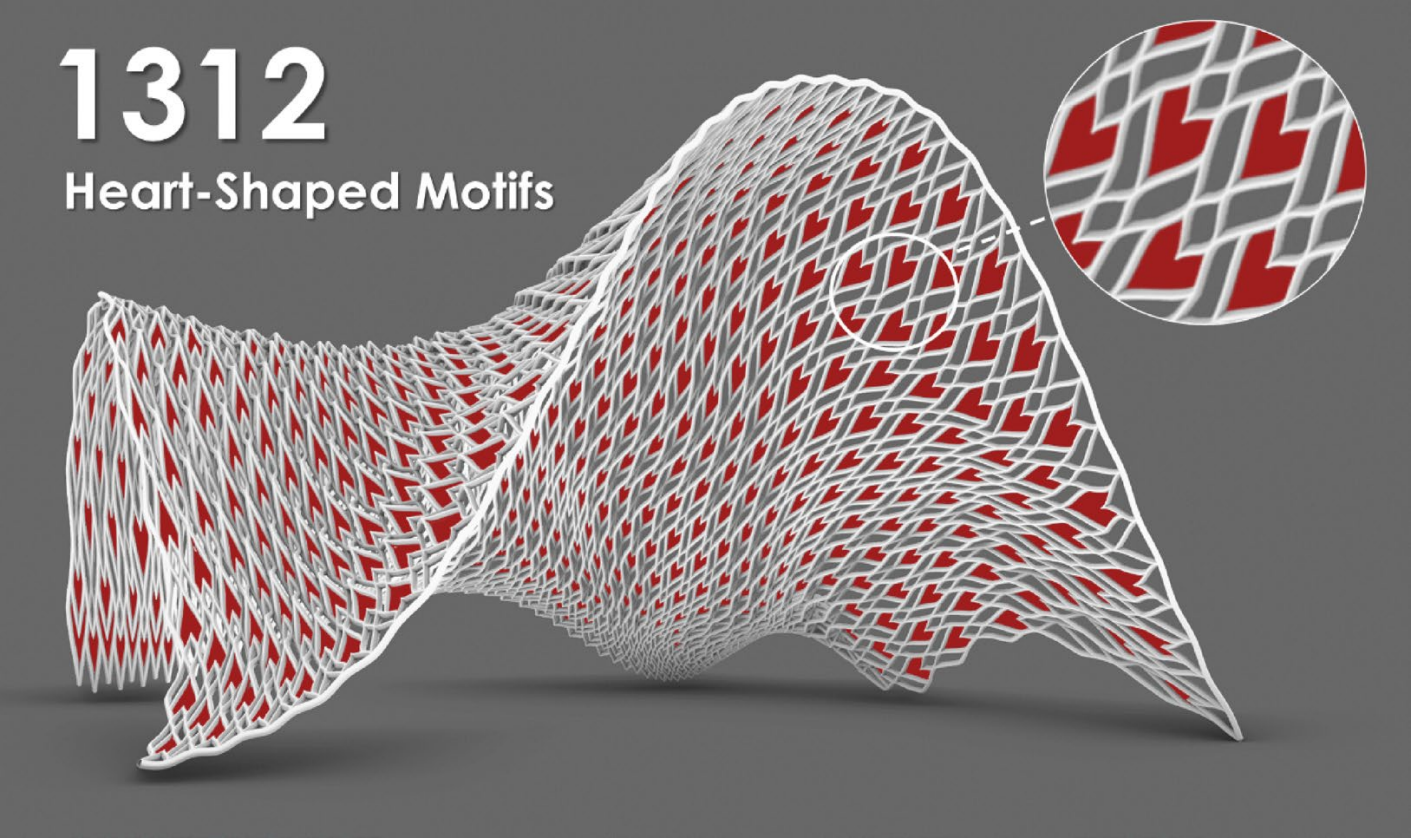
1312 heart-shaped motifs hidden within, symbolizing eternal love in the local culture.

The “Weaving Love” pavilion (Fig. 4a) is a 3D-printed metal structure within the sky garden of the New Immigration Headquarters of Hong Kong in Tseung Kwan O Hong Kong, China. Designed as a photographic backdrop for weddings, the pavilion combines complex geometric forms (Fig. 4b) with structural functionality. The pavilion features 1312 heart-shaped motifs (Fig. 5), symbolizing eternal love in the local culture, and was fabricated using WAAM technology. This advanced manufacturing technique enables the creation of complex, freeform structures with minimal material waste.

The pavilion’s design concept drew inspiration from the visual characteristics of a bridal veil. Its continuous, undulating surface creates an immersive spatial experience, designed to integrate with the surrounding landscape. The design process was highly iterative, with over 10 design schemes explored before finalizing the concept. Key considerations included structural stability, printability, material efficiency, and the integration of light and shadow play to enhance the romantic atmosphere of the space.

### Stages of design development

**Fig. 6:**
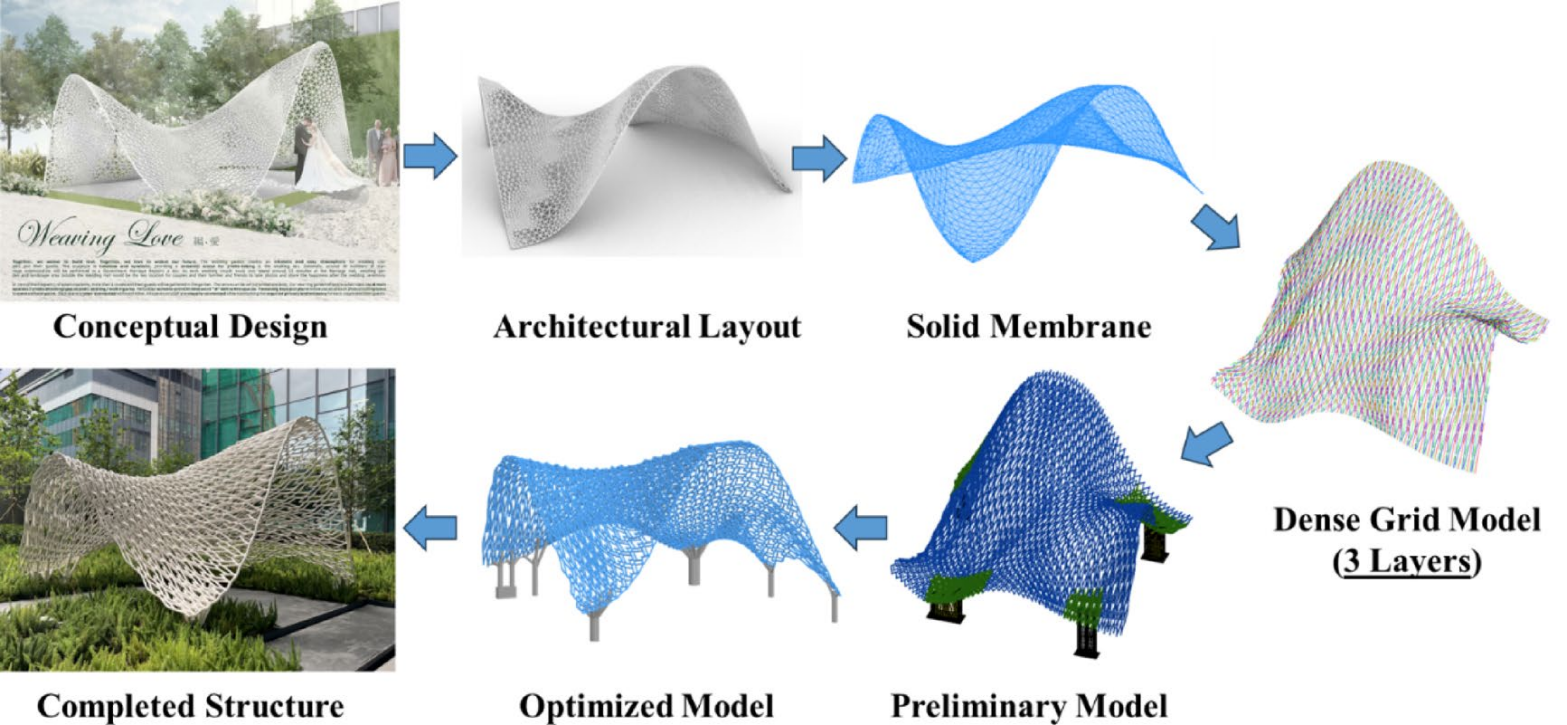
Design development flow diagram.

The design development for the 3D-printed structure involved several stages, as illustrated in Fig. 6. Each stage built upon the previous one, refining the concept into a structurally sound and visually striking design:

- *Conceptual design*: The initial phase focused on capturing the essence of a bridal veil—its softness, movement, and symbolism. Sketches and digital renderings explored various forms and motifs, including waves, curves, and geometric patterns.
- *Architectural layout*: The conceptual design was translated into an architectural layout, defining the spatial arrangement and proportions of the pavilion. This stage ensured that the structure would harmonize with its surroundings while meeting functional requirements.
- *Solid membrane*: In this stage, the architectural layout was transformed into a solid membrane model, which served as the foundation for structural and aesthetic refinement. Sophisticated finite element analysis using shell elements was employed to evaluate stress intensity within the structure, allowing for the confirmation of appropriate membrane thickness. This step emphasized the continuity of the form, ensuring that the pavilion’s flowing lines and intricate details were preserved.
- *Dense grid model*: Imitating the three-layer weaving structure of the bridal veil, a detailed dense grid model was created to represent the structure’s geometry at a high resolution. Also, the outer and inner skins act as compression‑ and tension‑membranes; the middle web transfers shear and braces the shell. This tri‑layer arrangement doubles global rigidity, keeps members within the WAAM bead’s optimal 42° print‑slope, and preserves the veil‑like transparency the concept demands—making it the empirically proven optimum for strength, printability, and aesthetics. This model was designed using the second-order direct analysis method through a nonlinear computer analysis approach, considering both ultimate and serviceability limit states. This model allowed the team to analyse stress distribution, optimize material use, and refine the design for fabrication.
- Preliminary model: To reduce fabrication workload—including print time, segment handling, and weld count—while preserving the essential load-carrying paths identified in the Dense Grid Model, the tri-layer lattice was simplified into a single structural layer composed of larger members. Only members that carried significant forces were retained, while low-stress members were removed. This reduction strategy effectively balances structural performance with fabrication practicality, minimizing material consumption and construction complexity without compromising stability or aesthetic integrity.
- Optimized model: The final optimized model was developed with the advanced structural design and study the most optimal supporting locations, which enabled a significant reduction in material usage while maintaining structural integrity. Member sizes were refined to be smaller and more efficient, based on stress distribution and load paths identified in earlier models. Additionally, the design of footing supports was carefully considered by evaluating various support locations to ensure proper load transfer and overall stability. This optimization process was essential not only for enhancing structural performance but also for minimizing environmental impact and fabrication complexity, aligning with the project’s sustainability and aesthetic goals.

### Topology optimization and material efficiency

**Fig. 7:**
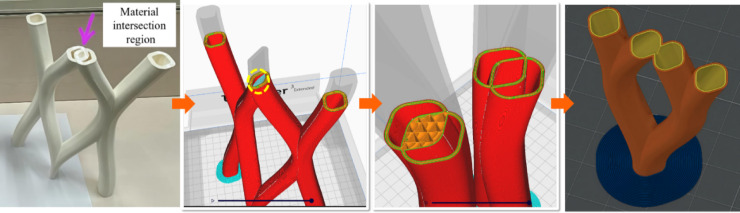
Topology optimization for the intersections at joints for material efficiency.

Topology optimization played an important role in the design process, enabling the project to achieve both cost-effectiveness and environmental sustainability. By analysing stress distribution and load paths, areas where material could be reduced without compromising structural performance were identified. To effectively address the intersections at joints, topology optimization was applied specifically to enhance material efficiency at these critical points, as illustrated in Fig. 7. This approach not only reduced material waste but also enhanced the pavilion’s visual lightness.

### Parametric modelling and computational structural analysis

**Fig. 8:**
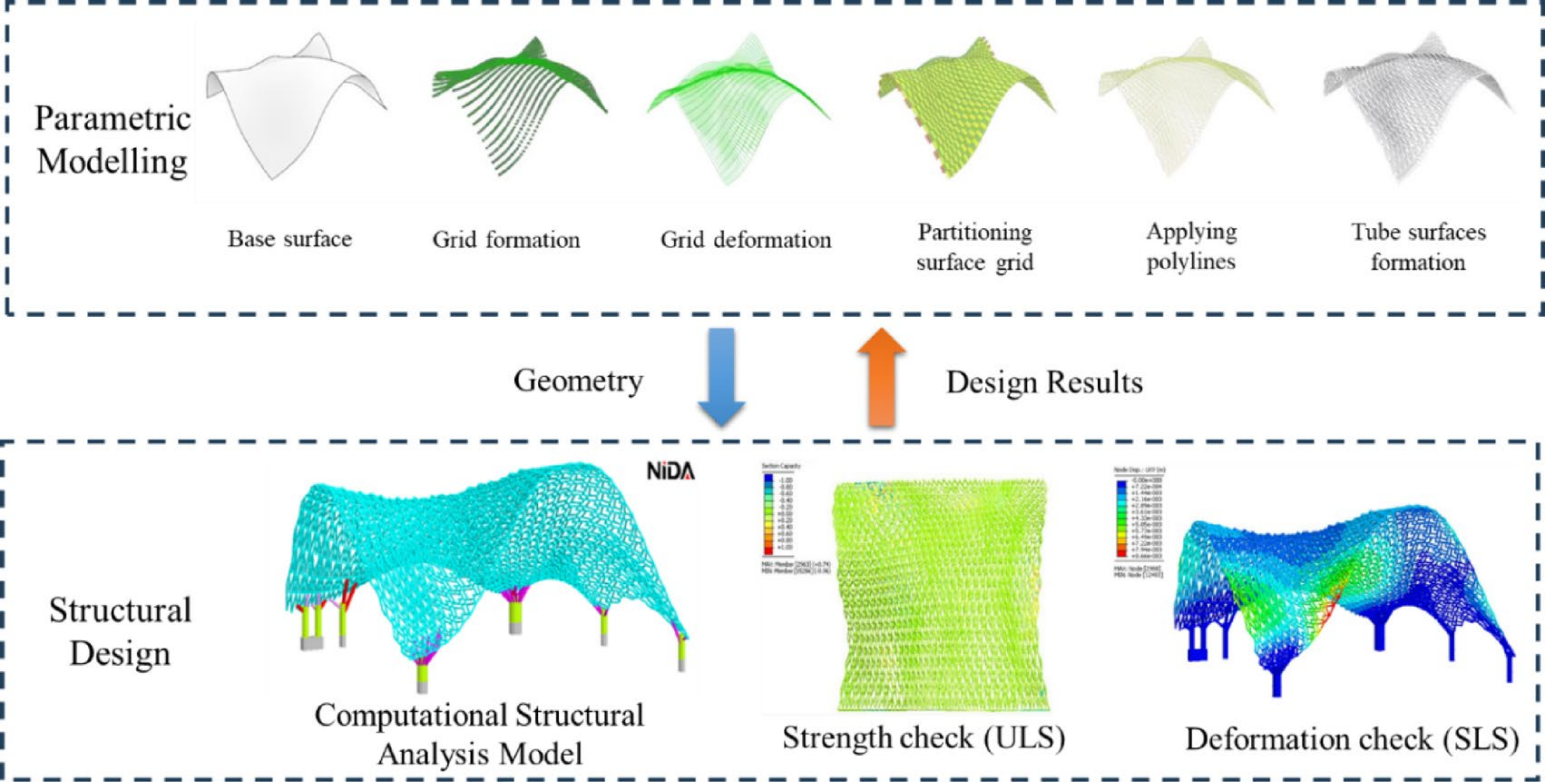
Interactive design workflow using parametric modelling and computational structural analysis.

Parametric modelling and computational structural analysis were utilized to optimize the design across various pattern options, as illustrated in Fig. 8. The design model generated through parameter modelling was analysed using the advanced software—NIDA Professional version 10^32^, which supports rigorous nonlinear simulations under multiple load scenarios, including dead load, live load, wind loads and temperature effects. Based on the structural design results, the parametric model was iteratively modified to meet structural performance requirements. These simulations also informed key design decisions regarding wall thickness, curvature, and joint connections, ensuring that the final design is both structurally sound and durable.

### Light and shadow interaction

The interaction between light and shadow was a key consideration throughout the design process. The design team carefully studied how sunlight would interact with the pavilion’s intricate patterns and flowing form, aiming to create a dynamic and romantic atmosphere. Techniques such as perforations, varying surface textures, and strategic placement of heart-shaped motifs were used to enhance the interaction of light and shadow within the space.

**Fig. 9:**
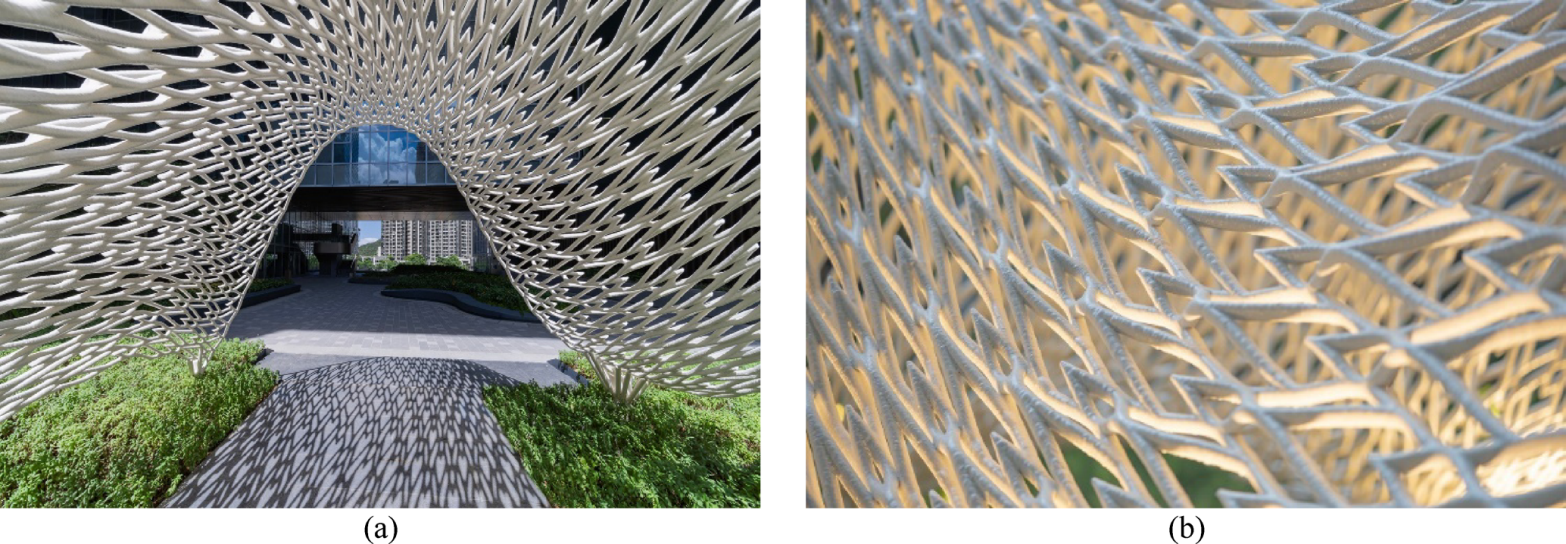
The interaction between light and shadow: (**a**) natural sunlight casting intricate shadows on the ground; (**b**) an integrated lighting accentuates the pavilion.

For example:

- During the day, sunlight filters through the heart-shaped motifs, casting intricate shadows on the ground (Fig. 9a).
- At night, integrated lighting accentuates the structure’s curves and patterns, creating a warm and inviting ambiance (Fig. 9b).

## Geometric fine-tuning: deposition rate, surface texture and printability

The geometric design of the “Weaving Love” pavilion required meticulous fine-tuning to ensure both aesthetic appeal and structural integrity. Key parameters such as deposition rate, surface texture, printability, and printing path were carefully optimized during the WAAM process, where the digital printing simulation method was adopted. These parameters collectively ensured the feasibility of translating a complex digital design into a physically robust and visually striking structure.

### Deposition rate optimization

The deposition rate directly impacts build time, material efficiency, and resolution. For “Weaving Love”, balancing speed and precision was critical. Adjustments to wire feed rate and torch travel speed ensured uniform deposition while preserving intricate patterns.

### Surface texture and bead geometry

The wire feed angle (optimized between 20° and 60° from vertical) governed bead geometry and surface finish. Angles outside this range caused rippling or instability, underscoring the need for precise control to achieve smooth aesthetics and structural reliability.

**Fig. 10:**
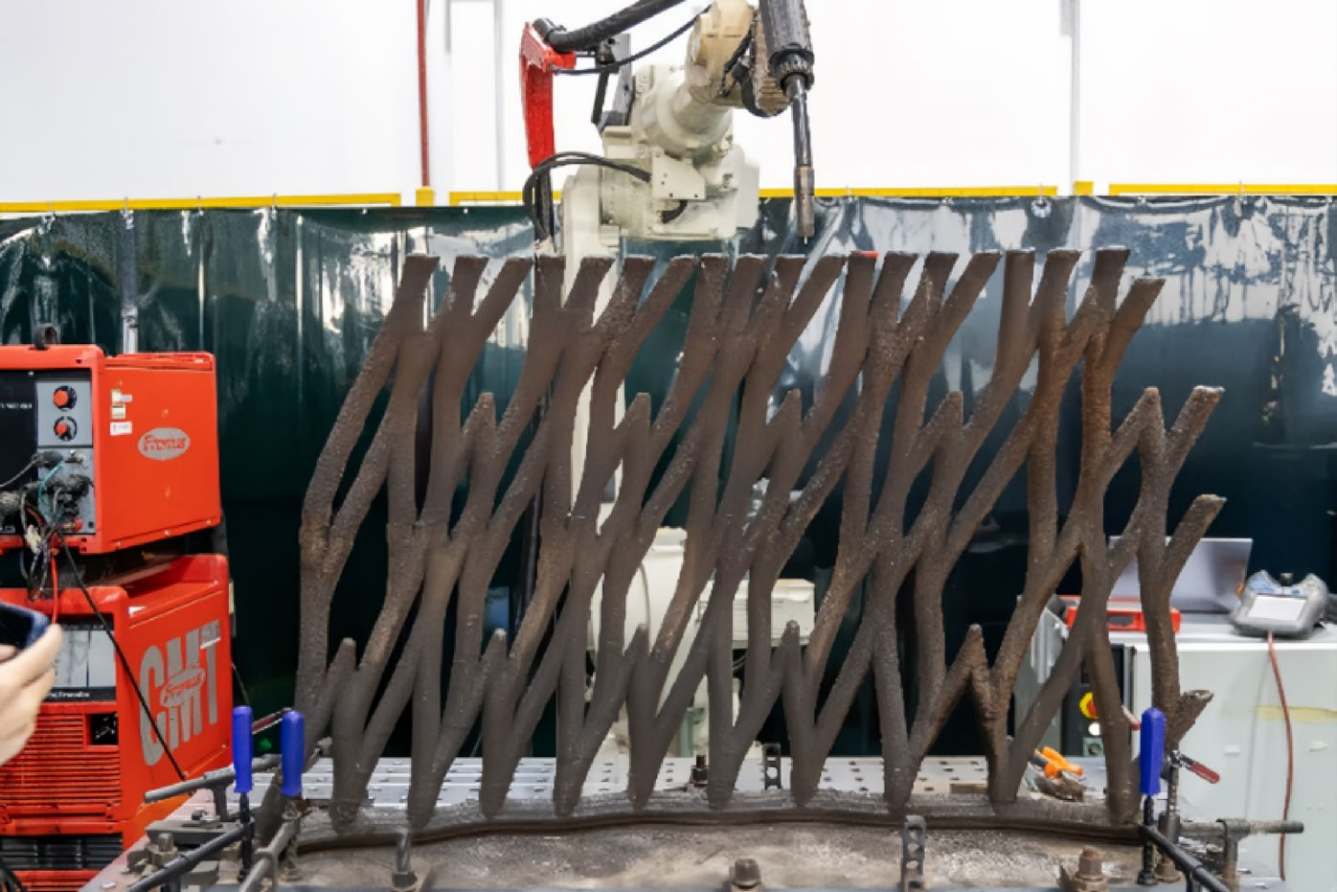
Printed samples during development phase.

To ensure the quality of the surface texture and deposition rate, several printed samples were produced and tested during the development phase (Fig. 10). These samples were used to evaluate the surface finish, bead geometry, and overall printability of the material. The results from these tests informed the final adjustments to the WAAM process, ensuring that the surface texture met the desired aesthetic standards, and that the deposition rate was optimized for both speed and quality.

### Printability and layer-by-layer deposition

**Fig. 11:**
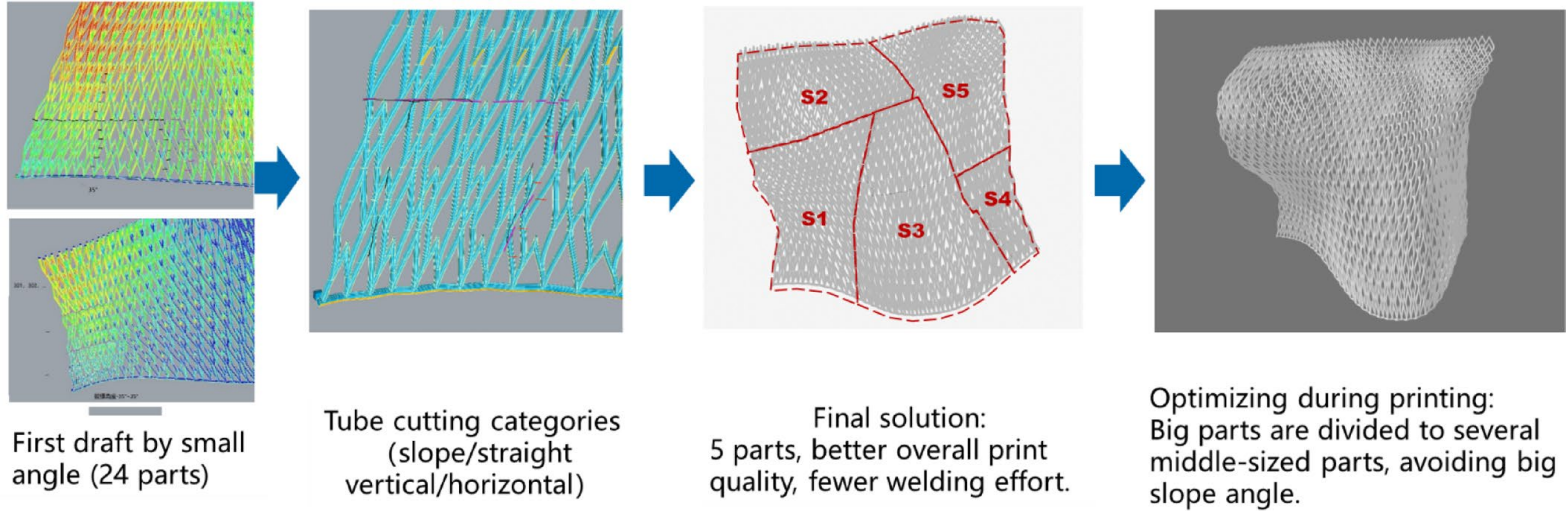
Segmentation scheme for optimal printing quality and structural integrity.

Layer-by-layer deposition was managed through robotic arms and real-time torch adjustments. 3D scanning validated dimensional accuracy at each stage, ensuring alignment with the digital model. To address challenges in printing complex geometries, segmentation analysis divided the pavilion into manageable sections. Key strategies included minimizing print angles within 42° to prevent overhangs and material dripping, alongside optimized segmentation schemes that balanced structural integrity with fabrication feasibility (Fig. 11). This ensured the intricate heart-shaped motifs and fluid forms could be accurately reproduced.

### Printing path optimization

**Fig. 12:**
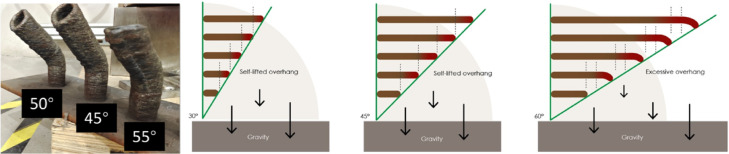
Print slope angle test, demonstrated the optimal printing geometry at < 45°

The printing path, the trajectory of the deposition tool—was critical for navigating the pavilion’s intricate geometry shape. A 45°-50°-55° slope angle printing test evaluated wall quality and optimized parameters for challenging geometries (Fig. 12). Key refinements included adjusting wall thickness to 4 mm for stability, conducting trial prints and weld tests to verify deposition consistency, and fine-tuning torch speed and wire feed rate to mitigate thermal distortion. Algorithmic path optimization ensured: (a) minimal abrupt directional changes to reduce residual stress; (b) continuous deposition for uniform layer adhesion; and (c) collision avoidance between the robotic arm and printed segments. Together, these parameters enabled the creation of a complex, large-scale metal structure with minimal post-processing, showcasing potential of WAAM for architectural innovation.

## Structural analysis and design

**Fig. 13:**
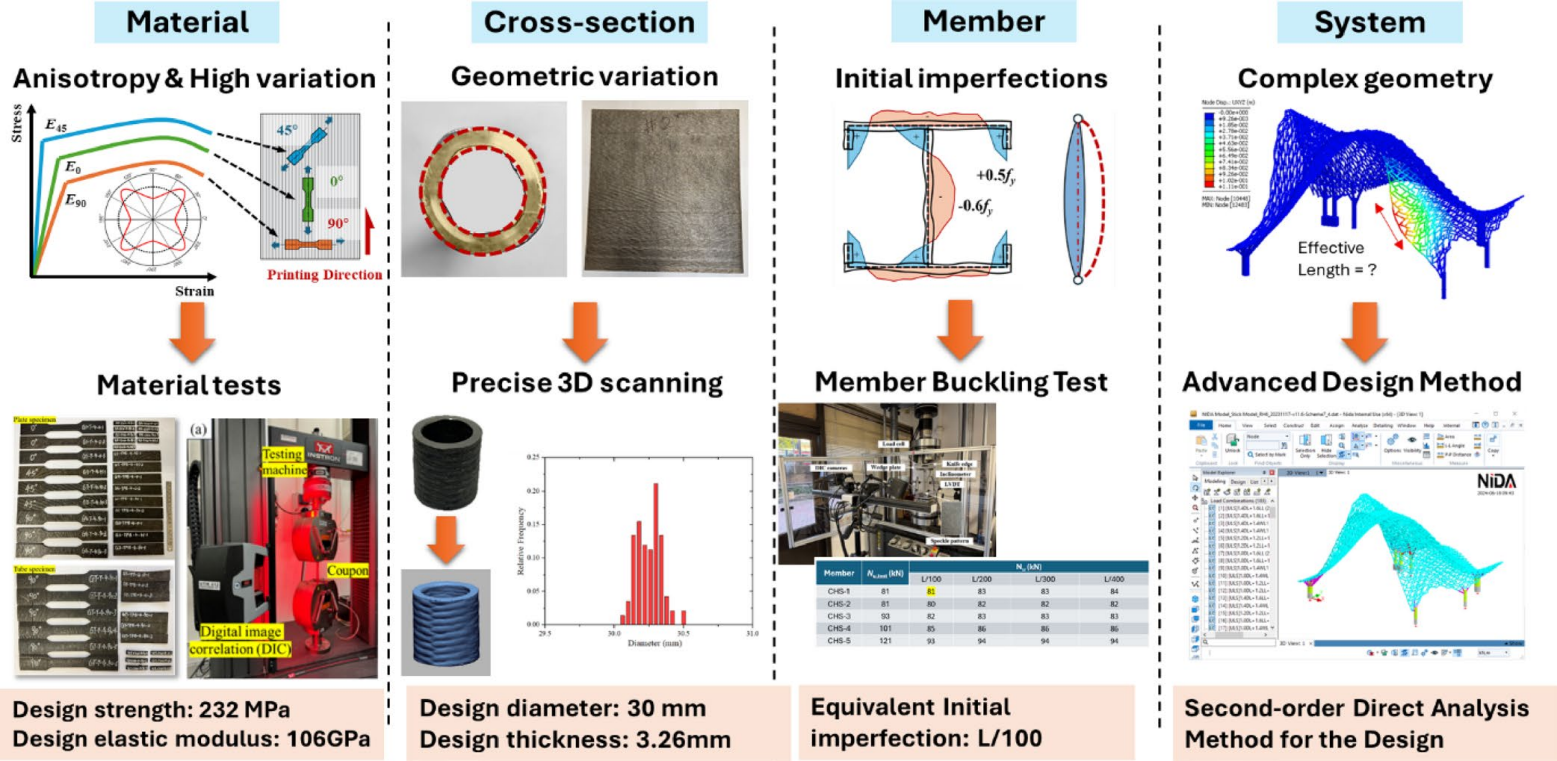
Structural design challenges and corresponding solutions.

The structural design of the Weaving Love pavilion, measuring 5.3 m × 4.8 m × 3.8 m and weighing over 2000 kg, required careful integration of aesthetic appeal with functional performance under environmental loads, including wind pressures of 2.1–2.9 kPa and temperature variations from + 35 °C to − 25 °C. Key design challenges (Fig. 13) included: (1) material anisotropy and variability, addressed by adopting mechanical properties at 90° to the printing direction and applying a 99.7% statistical acceptance ratio; (2) geometric variations in cross-sections and plate thickness, resolved through precise 3D scanning and conservative use of lower-bound measured values; (3) unknown initial imperfections, such as geometric initial out-of-straightness and residual stresses, tackled by physical testing of members with varying slenderness ratios to determine equivalent imperfections; and (4) complex geometry that rendered conventional linear analysis methods ineffective, necessitating the use of second-order direct analysis for accurate stability assessment. Through these targeted strategies, the pavilion was successfully designed in accordance with Hong Kong’s structural design standards.

### Material testing and verification

**Fig. 14:**
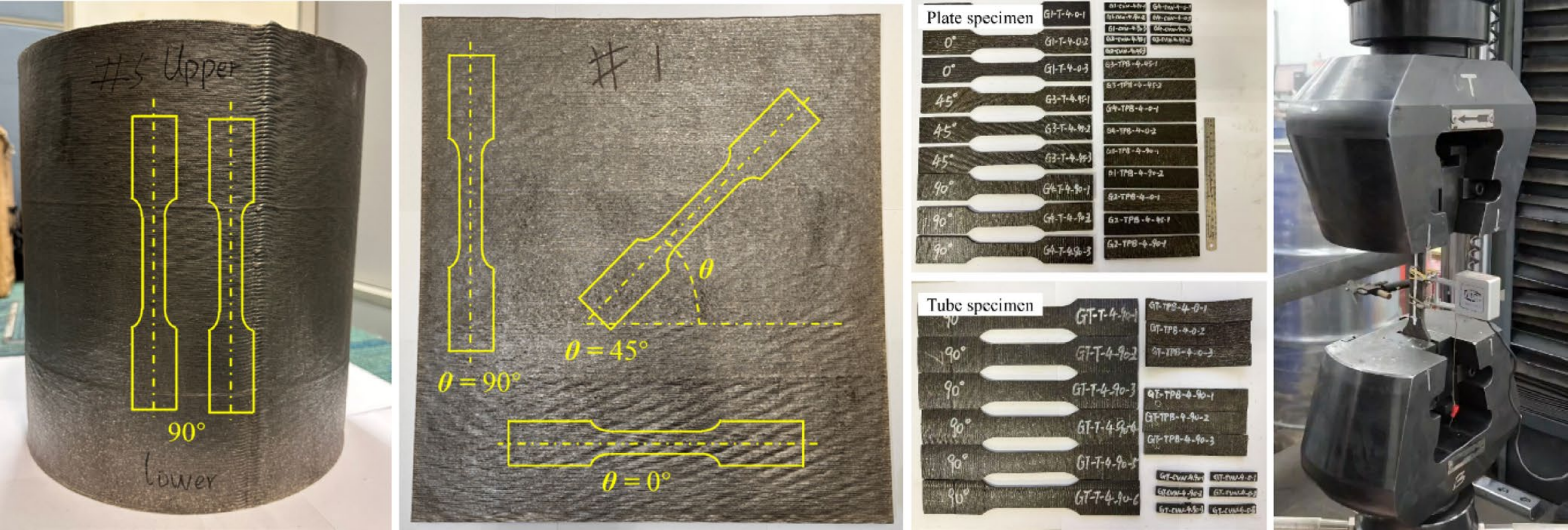
Tensile test specimens.

To ensure safety and performance, stainless steel 308L (Class2:SS308L) was selected for its corrosion resistance and WAAM compatibility. The welding wire JQWG308L (S308L) adhered to international standards (ISO 14,343:2009(E)^34^) for pressure equipment gas protection. Chemical composition testing ensured uniformity, while tensile tests confirmed a design strength of 232 MPa (Fig. 14), aligning with yield strength and ultimate tensile strength results as shown in Table 1. The test yield strengths of the specimens are summarized and the lower bound with a 95% acceptance ratio of the yield strength is determined as 256 MPa. The design strength is obtained by applying material factor 1.1 to it, and it is calculated as 232 MPa.

**Table 1:** Tensile test results.

Specimen	Printing direction (°)	Elastic modulus (MPa)	0.2% proof strength (MPa)	1.0% proof strength (MPa)	Ultimate strength (MPa)	Elongation (%)
G1-T-4-0-1	0	1.73 × 10^5^	315	332	527	32
G1-T-4-0-2	0		308	330	522	34
G1-T-4-0-3	0		320	338	536	31
G3-T-4-45-1	45	1.23 × 10^5^	296	320	481	57
G3-T-4-45-2	45		305	321	468	58
G3-T-4-45-3	45		304	325	483	57
G4-T-4-90-1	90	1.06 × 10^5^	289	328	492	37
G4-T-4-90-2	90		300	330	490	32
G4-T-4-90-3	90		301	335	492	30
GT-T-4-90-1	90	1.35 × 10^5^	292	322	489	33
GT-T-4-90-2	90		263	320	490	34
GT-T-4-90-3	90		264	309	473	33
GT-T-4-90-4	90		267	315	477	34
GT-T-4-90-5	90		274	323	493	34
GT-T-4-90-6	90		249	322	493	35

**Fig. 15:**
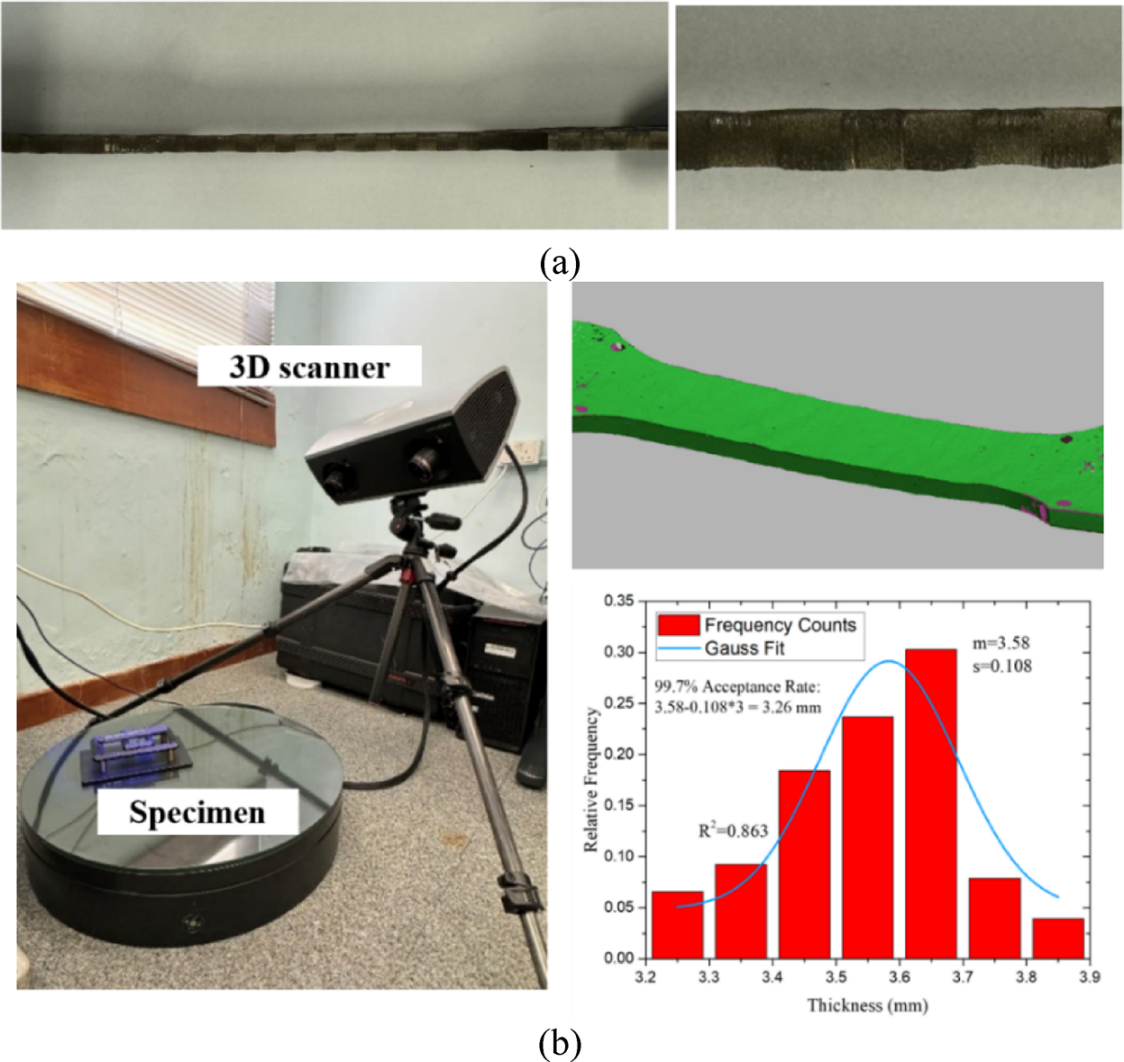
Specimen thickness analysis: (**a**) Varied thickness of the WAAM steel plate; (**b**) 3D scanning and statistical analysis results.

Dimensional consistency tests revealed a mean thickness of 3.58 mm (standard deviation: 0.108 mm), closely matching the design specification of 3.26 mm, with a 99.7% acceptance ratio (Fig. 15). Advanced 3D scanning verified compliance with tolerances, ensuring structural reliability.

### Structural analysis, design and verification

**Fig. 16:**
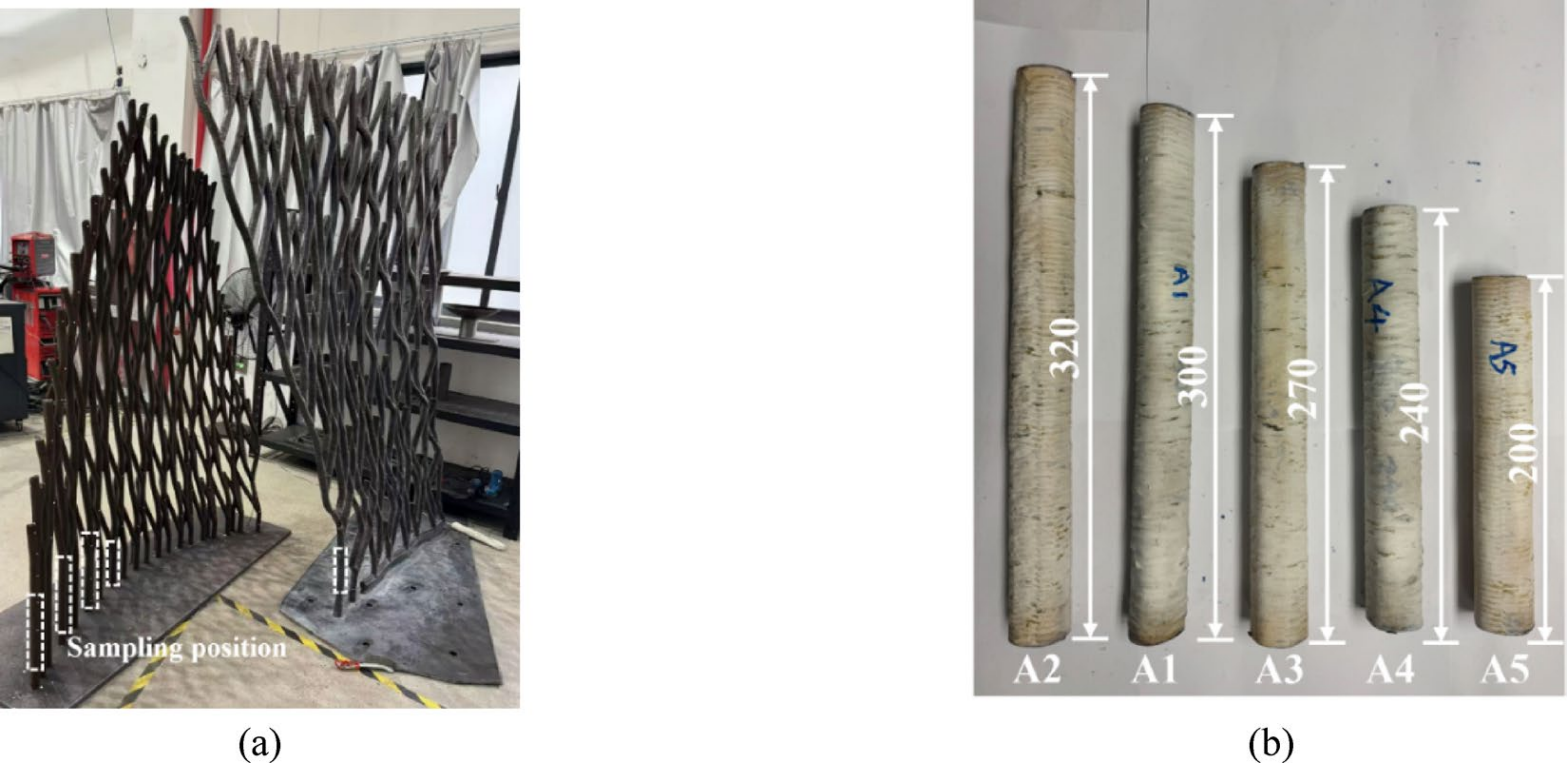
Compression test: (**a**) sampling position; (**b**) test specimens.

Given the geometric undulation and irregularity of the structure, a performance-based approach was adopted for structural design. The structural team performed a second-order direct analysis^29,35–38^, which incorporated both P-Δ and P-δ effects, as well as equivalent initial imperfections (combing initial member out-of-straightness and residual stresses). Based on physical testing of sampled members extracted from the structure (see Fig. 16), the equivalent initial imperfection for individual members was taken as L/100, as validated by the test results summarized in Table 2. Additionally, global frame imperfections were assumed to be height/200.

**Table 2:** Member imperfections and buckling loads.

Specimen	Length L (mm)	Maximum geometric imperfection (mm)	N_u, test_ (kN)	N_u, pre._ (kN)	N_u, pre._/N_u, test_ (kN)
A1	300	1.55 (*L*/194)	81.2	79.2	0.98
A2	320	0.91 (*L*/352)	81.9	79.3	0.97
A3	270	0.73 (*L*/370)	92.8	85.6	0.92
A4	240	1.04 (*L*/231)	100.9	86.3	0.86
A5	200	0.57 (*L*/351)	120.5	99.1	0.82

**Fig. 17:**
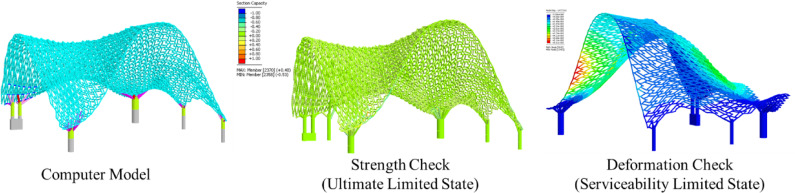
Computational structural analysis using second-order direct analysis.

An advanced computational structural analysis tool, NIDA Professional version 10^32^, was used to predict the behaviour of the structure under various load scenarios, including dead load, live load (0.75 kPa), temperature effects (− 25℃ to + 35℃), and wind loads of 2.1 kPa to 2.9 kPa from different directions. These loadings were determined in accordance with the Hong Kong wind code^39^ and structural load code^40^. Specifically, the wind loads were applied in eight horizontal directions and two vertical directions. The member load capacity and deflection were assessed under the ultimate limit state (ULS) and serviceability limit state (SLS), respectively (Fig. 17).

## Printing fabrication

The fabrication of the “Weaving Love” pavilion involved rigorous planning and execution, leveraging advanced WAAM technology. The process was carefully monitored and controlled to ensure dimensional accuracy, structural integrity, and aesthetic excellence. Below is a detailed overview of the fabrication process.

### Robot specifications and setup

**Fig. 18:**
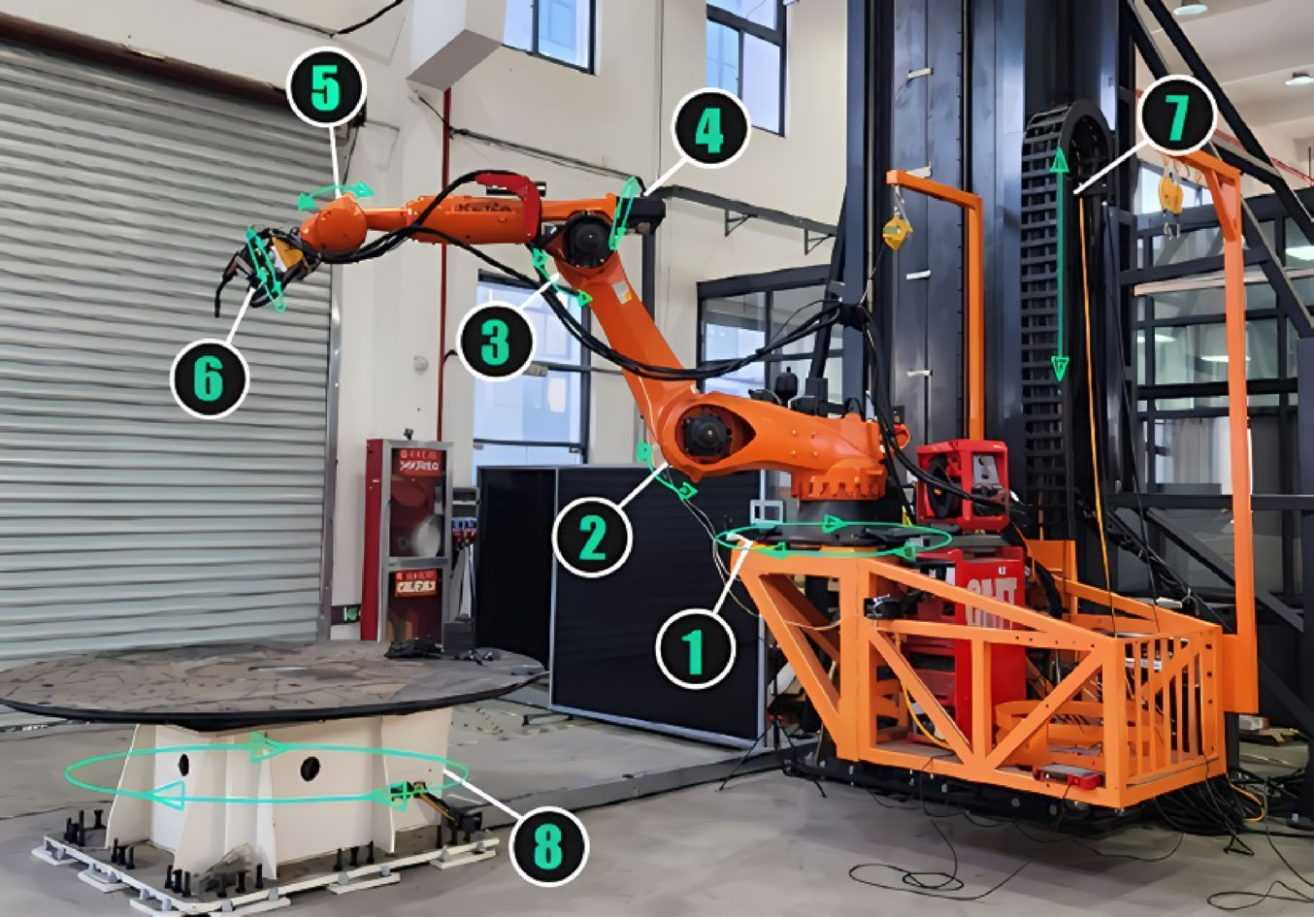
Eight-axis robotic printing station.

The fabrication process utilized two robot stations, each equipped with an eight-axis rotational platform. These stations were configured to handle the complex geometries of the pavilion (Fig. 18). The printable dimensions of the robot stations were as follows:

- Robot station 1(RS-R1): 3.1(L) × 3.1(W) × 6.6 m(H)
- Robot station 2(RS-L2): 4.0(L) × 2.0(W) × 2.0 m(H)

These dimensions allowed for the creation of large-scale components while maintaining precision and control.

### Printing simulations and preparation

**Fig. 19:**
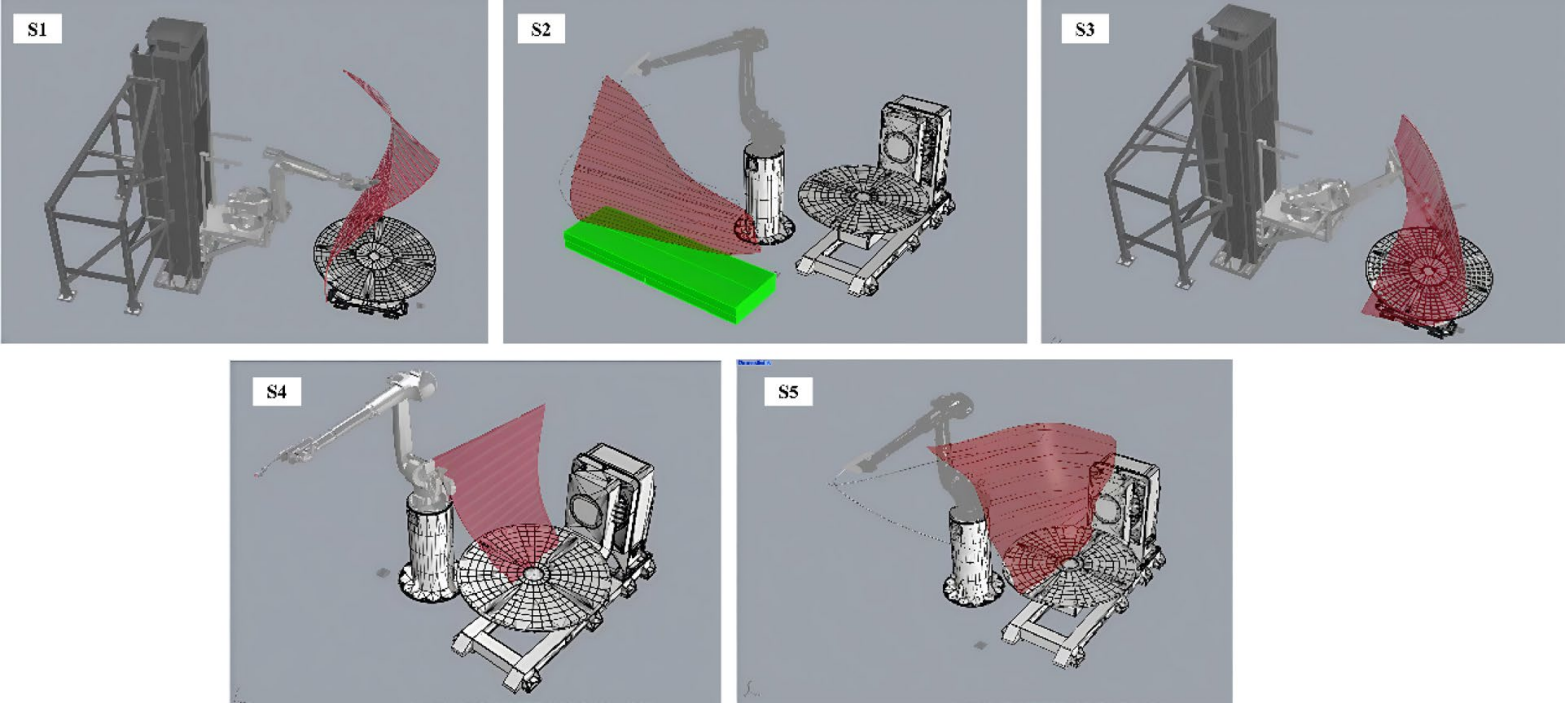
Printing simulation for S1 to S5.

Prior to actual printing, extensive simulations were conducted to optimize the printing parameters and ensure the feasibility of the design. The simulations involved multiple segments (S1 to S5), each focusing on different aspects of the printing process (Fig. 19).

### On-site monitoring and control

**Fig. 20:**
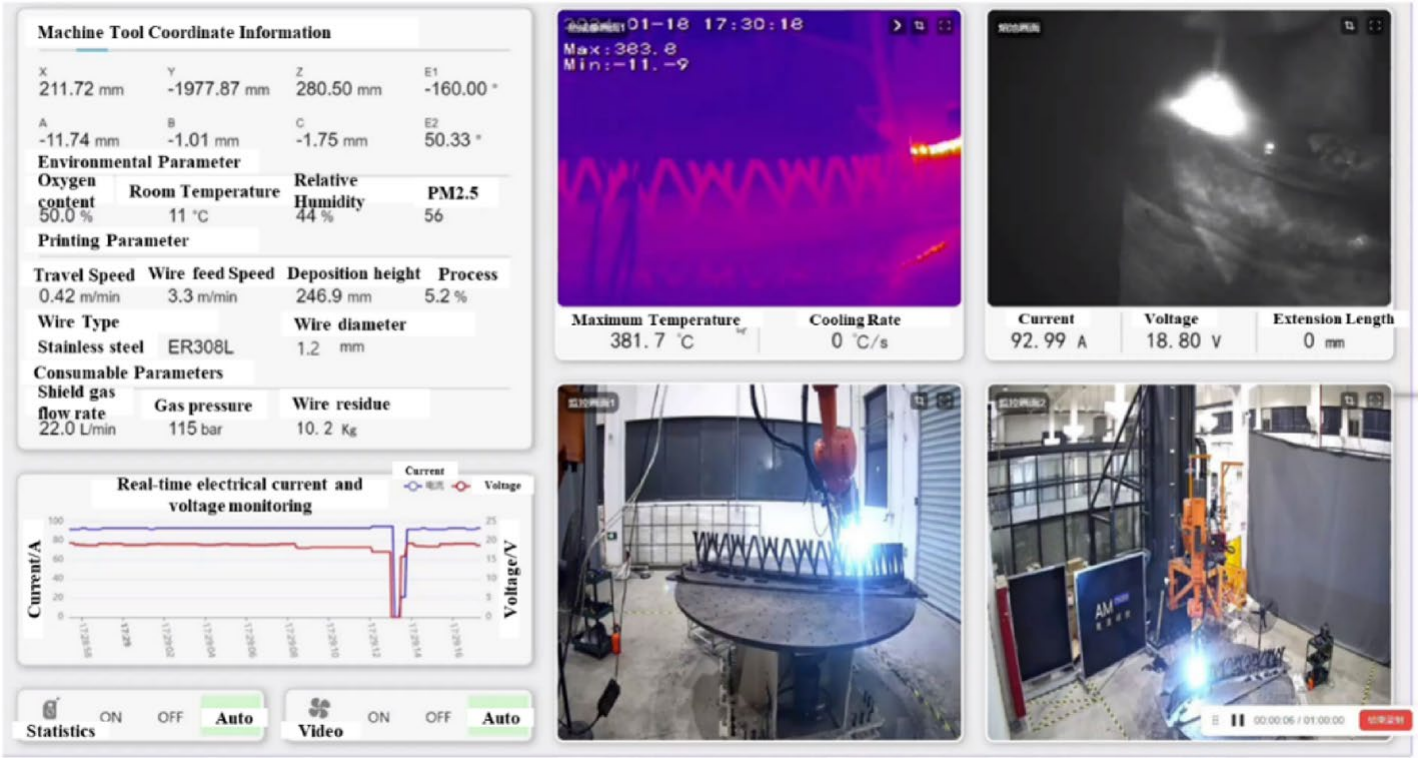
Real-time monitoring and control.

**Fig. 21:**
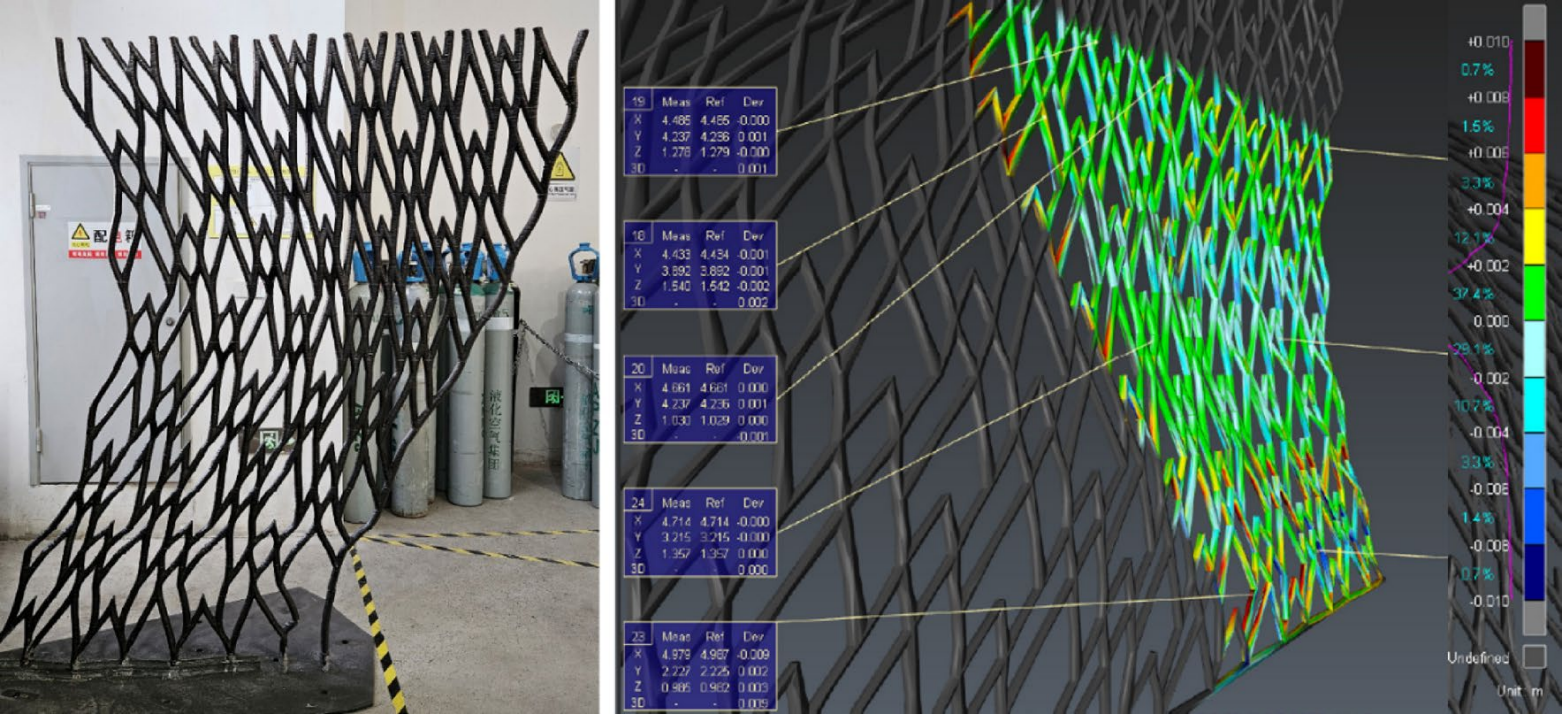
Intermediate 3D scanning.

During the actual printing process, real-time monitoring was implemented to control and adjust printing parameters as needed (Fig. 20). Key aspects monitored included:

- Environmental conditions: Humidity levels were maintained at 44% to 50%, and temperatures were monitored to ensure the printing conditions.
- Mechanical parameters: Voltage, current, feed and travel speed were continuously adjusted to maintain dimensional integrity and surface quality.
- Material and deposition: Stainless steel ER308L wire with a diameter of 1.2 mm was used. The deposition rate was carefully controlled to ensure uniform layering and structural strength.
- Dimensional integrity: 3D scan was employed at various stages to verify dimensional accuracy and ensure alignment with the digital model (Fig. 21).

### Segment fabrication and assembly

**Fig. 22:**
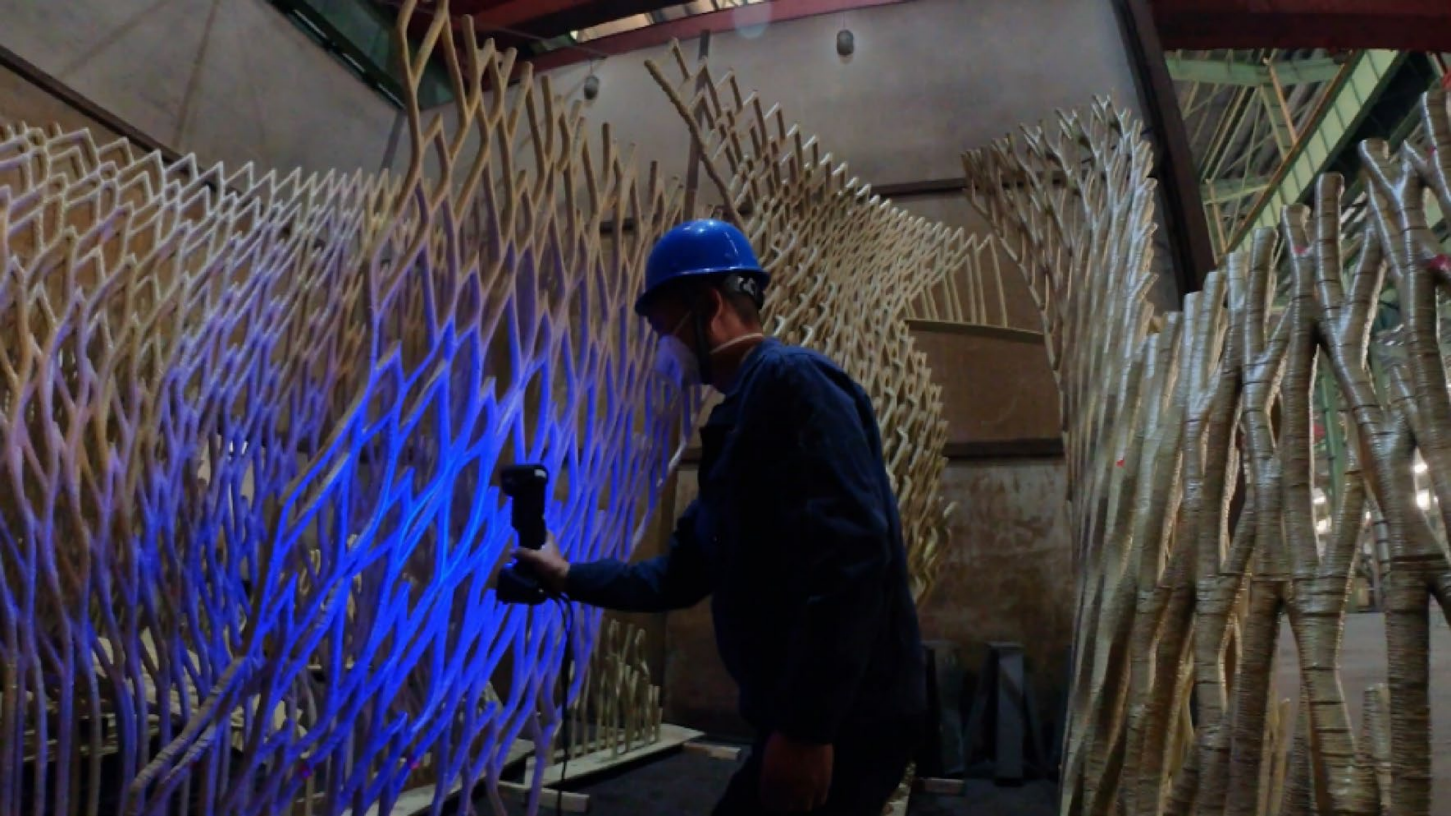
3D scans and visual inspections.

The pavilion was divided into manageable segments to facilitate printing and assembly (Fig. 11). Each segment was printed and post-processed individually, ensuring dimensional accuracy and surface quality (Fig. 22).

**Fig. 23:**
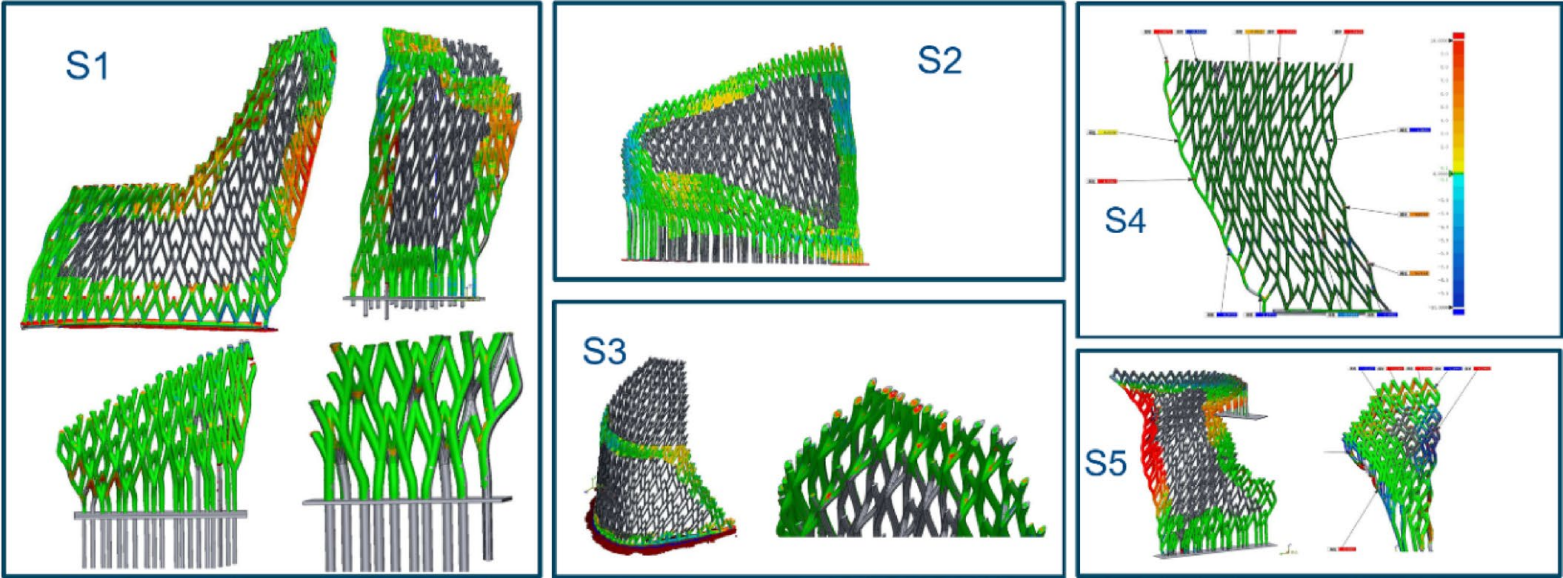
3D scanning of each segment.

**Fig. 24:**
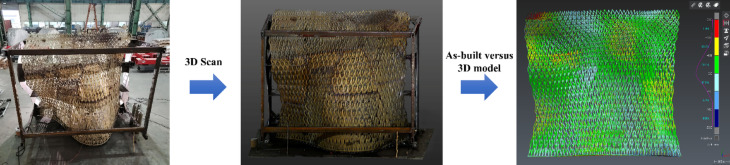
Post-assembly 3D scan to ensure overall dimensional integrity.

Key steps included:

- Segmentation analysis: The pavilion was segmented to minimize print angles and ensure structural integrity.
- Post-processing: Each segment underwent grinding, sandblasting, and rustproofing to ensure surface quality and durability.
- Assembly: Prior to the assembly, each segment was 3D scanned to ensure assembly accuracy (Fig. 23). The segments were then assembled using a custom metal frame that followed the curvature of the structure (Fig. 24). Over 600 weld joints were completed using manual MIG and TIG welding techniques in the factory, followed by weld inspections and load tests to confirm structural integrity.

## On-site preparation and installation

**Fig. 25:**
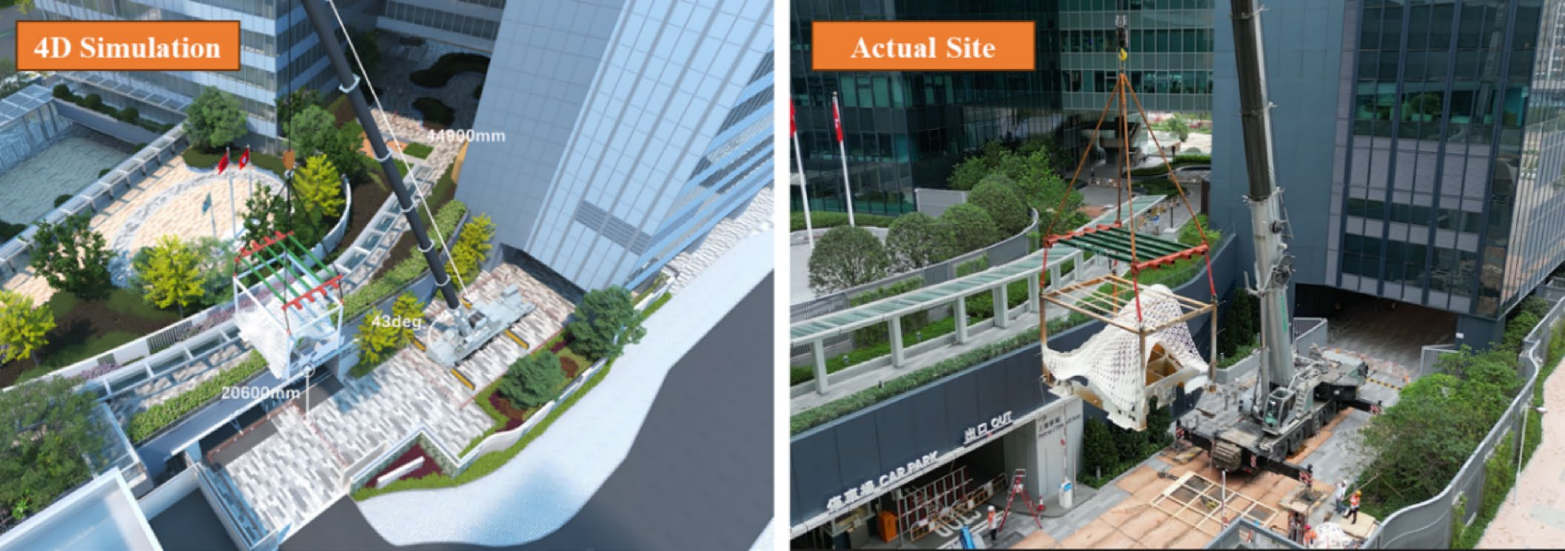
4D simulation (Virtual) and Actual Single-day installation (Reality).

Comprehensive planning and execution of the pavilion’s installation was the final step of this project. Through advanced simulation, risk assessment, and safety coordination, the project team ensured optimal site safety and achieved a single-day installation with minimal disruption. Key aspects included:

- 4D Simulation and planning: a 4D simulation was conducted to visualize the installation process, optimize the schedule, and identify potential conflicts (Fig. 25). This allowed for proactive adjustments to the installation sequence, ensuring efficient use of resources and minimizing disruptions to building operations.
- Risk assessment and treatment: A comprehensive Risk Assessment and Treatment was performed to identify and mitigate potential hazards. Key risks, such as structural stability during installation and site safety, were addressed through detailed mitigation strategies.
- Pre-work safety meetings: Numerous pre-work safety meetings were held with the installation team to review safety protocols, emergency procedures, and responsibilities. These meetings ensured all personnel were aligned with safety measures and contingency plans.
- Installation: The pavilion was installed within one day, leveraging meticulous planning and coordination. The large structure was transported to the site fully assembled, allowing for a straightforward lift and installation to minimized the disruption.

## Achievement and future implications

The project achieved a 52% reduction in construction time, 67% cost savings, and 80% less material waste, based on a quantitative comparison between WAAM and conventional Computer Numerical Control (CNC) machining. Detailed calculations and methodology are provided in Appendix A. Key technical milestones included the successful 42° slope angle optimization, segmentation strategies for complex geometries, and rigorous material validation—all critical to balancing aesthetics, durability, and structural safety. A post-installation 3D scan was conducted for quality assurance, revealing that over 98.9% of the as-built point cloud data—after noise removal—fell within a ± 10 mm deviation from the design model. 

## Discussion and conclusion

This pioneering engineering project sets a precedent for the application of 3D metal printing in constructing geometrically complex structures, which are otherwise unfeasible using conventional steel manufacturing methods (e.g., hot rolling). The successful completion of the project is attributed to several key factors. First, the collaboration mode of “Government-University-Industry” facilitates the transformation of advanced printing technique into a real-world application. Second, optimization methods were employed during both the design and construction stages. As a highly customized manufacturing technique, 3D metal printing benefits significantly from optimization of the printing path, deposition rate, and geometry, which maximizes its performance and potential. Third, the project demonstrates the feasibility of applying an existing design methodology, namely, the second-order direct analysis method, to 3D-printed metal structures. Finally, a segmental fabrication strategy, combined with 3D scanning for dimensional integration, was adopted as a viable alternative for manufacturing large-scale 3D-printed metal structures.

Despite these achievements, the project also faced several limitations. For instance, there is a lack of well acknowledged mechanical properties in current design codes. To ensure safety under conservative principles, the weakest mechanical properties obtained from tensile tests (within a defined acceptance ratio) were selected for use in the design. Additionally, determining optimal printing parameters and strategies is both time-consuming and costly. Therefore, to promote the widespread adoption of 3D metal printing in conventional construction, the development of a well-established design guideline is essential. This includes defining structural design parameters (e.g., mechanical properties) and standardizing printing strategies (e.g., deposition rate, path, and dwell time) for reference. In conclusion, this project—spanning architectural design, structural analysis, and construction—presents a feasible and effective approach for realizing 3D-printed metal structures. To extend its application to mainstream construction, the establishment of standardized design guidelines and procedures for 3D metal printing is imperative.

“Weaving Love” pavilion is more than just a technological achievement; it is a testament to the power of innovation and collaboration in pushing the boundaries of what is possible in construction. By embracing advanced technologies like WAAM and parametric modelling, the project team has created a structure that is not only visually stunning but also environmentally friendly and cost-effective.

## Supplementary Information

Below is the link to the electronic supplementary material.

Supplementary Material 1

## Data Availability

The datasets used and/or analysed during the current study are available from the corresponding author upon reasonable request.

## References

[1] Sefene, E. M. State-of-the-art of selective laser melting process: A comprehensive review. *J. Manuf. Syst.* **63**, 250–274 (2022).

[2] Hilpert, E., Hartung, J., Risse, S., Eberhardt, R. & Tünnermann, A. Precision manufacturing of a lightweight mirror body made by selective laser melting. *Precis. Eng.* **53**, 310–317 (2018).

[3] Feng, R., Tang, J.-P., Quach, W.-M., Yan, M. & Zhao, J. Experimental study on additive manufactured 304L stainless steel tubular sections: Material properties and cross-sectional behavior. *Eng. Struct.* **273**, 115093 (2022).

[4] Zhang, L. et al. Mechanical metamaterials with negative Poisson’s ratio: A review. *Eng. Struct.* **329**, 119838 (2025).

[5] Gardner, L. Metal additive manufacturing in structural engineering—review, advances, opportunities and outlook. *Structures* **47**, 2178–2193 (2023).

[6] Meng, X. & Gardner, L. Hybrid construction featuring wire arc additive manufacturing: Review, concepts, challenges and opportunities. *Eng. Struct.* **326**, 119337 (2025).

[7] Evans, S. I. et al. A review of WAAM for steel construction—Manufacturing, material and geometric properties, design, and future directions. *Structures* **44**, 1506–1522 (2022).

[8] Hadjipantelis, N., Weber, B., Buchanan, C. & Gardner, L. Description of anisotropic material response of wire and arc additively manufactured thin-walled stainless steel elements. *Thin-Walled Struct.* **171**, 108634 (2022).

[9] Laghi, V. et al. Tensile properties and microstructural features of 304L austenitic stainless steel produced by wire-and-arc additive manufacturing. *Int. J. Adv. Manuf. Technol.* **106**(9–10), 3693–3705 (2020).

[10] Zhang, S., Zheng, B., Yao, J. & Shu, G. Test and constitutive modelling of wire arc additively manufactured stainless steel. *J. Constr. Steel Res.* **214**, 108474 (2024).

[11] Huang, C., Meng, X. & Gardner, L. Cross-sectional behaviour of wire arc additively manufactured tubular beams. *Eng. Struct.* **272**, 114922 (2022).

[12] Gardner, L., Li, J., Meng, X., Huang, C. & Kyvelou, P. I-section steel columns strengthened by wire arc additive manufacturing—Concept and experiments. *Eng. Struct.* **306**, 117763 (2024).

[13] Laghi, V., Palermo, M., Gasparini, G., Girelli, V. A. & Trombetti, T. Experimental results for structural design of wire-and-arc additive manufactured stainless steel members. *J. Constr. Steel Res.* **167**, 105858 (2020).

[14] Guo, X., Kyvelou, P., Ye, J., Teh, L. H. & Gardner, L. Experimental investigation of wire arc additively manufactured steel single-lap shear bolted connections. *Thin-Walled Struct.* **181**, 110029 (2022).

[15] Kyvelou, P., Spinasa, A. & Gardner, L. Testing and analysis of optimized wire arc additively manufactured steel trusses. *J. Struct. Eng.* **150**(3), 04024008 (2024).

[16] Kyvelou, P. et al. Mechanical and microstructural testing of wire and arc additively manufactured sheet material. *Mater. Des.* **192**, 108675 (2020).

[17] Chen, M.-T. et al. Mechanical behavior of austenitic stainless steels produced by wire arc additive manufacturing. *Thin-Walled Struct.* **196**, 111455 (2024).

[18] Meng, X., Weber, B., Nitawaki, M. & Gardner, L. Optimisation and testing of wire arc additively manufactured steel stub columns. *Thin-Walled Struct.* **189**, 110857 (2023).

[19] Kraus, M. et al. Geometric imperfections of additive manufactured members. *Eng. Struct.* **252**, 113596 (2022).

[20] Kyvelou, P., Huang, C., Li, J. & Gardner, L. Residual stresses in steel I-sections strengthened by wire arc additive manufacturing. *Structures* **60**, 105828 (2024).

[21] Gardner, L., Kyvelou, P., Herbert, G. & Buchanan, C. Testing and initial verification of the world’s first metal 3D printed bridge. *J. Constr. Steel Res.* **172**, 106233 (2020).

[22] Kyvelou, P., Buchanan, C. & Gardner, L. Numerical simulation and evaluation of the world’s first metal additively manufactured bridge. *Structures* **42**, 405–416 (2022).

[23] Wynne, Z. et al. Dynamic testing and analysis of the world’s first metal 3D printed bridge. *Case Stud. Constr. Mater.* **17**, e01541 (2022).

[24] Glashier, T., Kromanis, R. & Buchanan, C. Temperature-based measurement interpretation of the MX3D Bridge. *Eng. Struct.* **305**, 116736 (2024).

[25] Mai, D. S., Doan, T. K. & Paris, H. Wire and arc additive manufacturing of 308L stainless steel components: Optimization of processing parameters and material properties. *Eng. Sci. Technol.* **24**(4), 1015–1026 (2021).

[26] Jihong, Z., Han, Z., Chuang, W., Lu, Z. & Shangqin, Y. A review of topology optimization for additive manufacturing: Status and challenges. *Chin. J. Aeronaut.* **34**(1), 91–110 (2021).

[27] Laghi, V., Palermo, M., Bruggi, M., Gasparini, G. & Trombetti, T. Blended structural optimization for wire-and-arc additively manufactured beams. *Prog. Addit. Manuf.* **8**(3), 381–392 (2022).

[28] Laghi, V. & Gasparini, G. Explorations of efficient design solutions for wire-and-arc additive manufacturing in construction. *Structures* **56**, 104883 (2023).

[29] Liu, S.-W., Bai, R., Chan, S.-L. & Liu, Y.-P. Second-Order Direct Analysis of Domelike Structures Consisting of Tapered Members with I-Sections. *J. Struct. Eng.* **142**(5), 04016009 (2016).

[30] Liu, S.-W., Liu, Y.-P. & Chan, S.-L. Direct analysis by an arbitrarily-located-plastic-hinge element—Part 2: Spatial analysis. *J. Constr. Steel Res.* **103**, 316–326 (2014).

[31] Chan, S.-L. & Zhou, Z.-H. On the development of a robust element for second-order ‘non-linear integrated design and analysis (nida)’. *J. Constr. Steel Res.* **47**(1), 169–190 (1998).

[32] NIDA. Non-linear integrated design and analysis user’s manual, NAF-NIDA series, version 10 (2023).

[33] Mansor, M. S. M. et al. Integrated approach to wire arc additive manufacturing (WAAM) optimization: Harnessing the synergy of process parameters and deposition strategies. *J. Mater. Res. Technol.* **30**, 2478–2499 (2024).

[34] ISO 14343:2009(E). *Wire electrodes, strip electrodes, wires and rods for arc welding of stainless and heat resisting steels* (The International Organization for Standardization, 2009).

[35] Chan, S. L. & Zhou, Z. H. Second-order elastic analysis of frames using single imperfect element per member. *J. Struct. Eng.* **121**(6), 939–945 (1995).

[36] Zhang, H.-Y., Ho, G. W. M., Liu, S.-W., Chen, L. & Chan, S.-L. Advanced line-finite-element for lateral-torsional buckling of beams with torsion and warping restraints. *J. Constr. Steel Res.* **224**, 109103 (2025).

[37] Chen, L., Gao, W.-L., Liu, S.-W., Ziemian, R. D. & Chan, S.-L. Geometric and material nonlinear analysis of steel members with nonsymmetric sections. *J. Constr. Steel Res.* **198**, 107537 (2022).

[38] Liu, S.-W., Gao, W.-L. & Ziemian, R. D. Improved line-element formulations for the stability analysis of arbitrarily-shaped open-section beam-columns. *Thin-Walled Struct.* **144**, 106290 (2019).

[39] Code of Practice on Wind Effects in Hong Kong 2019 (The Building Department, Hong Kong, 2019).

[40] Code of Practice for Dead and Imposed Loads 2011 (2021 Edition) (The Building Department, 2021).

