# Supplementary material for: Design and fabrication of Hong Kong’s first constructional 3D-printed metal pavilion ‘Weaving Love’
Source: Sci Rep. 2025 Sep 26;15:32982. doi: 10.1038/s41598-025-17612-y (PMC12474970; doi:10.1038/s41598-025-17612-y)
Supplement: Supplementary file 1 — Supplementary Material 1 [file 41598_2025_17612_MOESM1_ESM.docx]

# **Appendix A. Quantitative Analysis of WAAM versus CNC Manufacturing**

A quantitative analysis of WAAM versus CNC manufacturing is provided in this appendix, where the material waste reduction, energy saving, manufacturing time reduction, operating cost saving, and embodied carbon reduction are calculated.

## ***Material Waste Reduction***

The expression of material waste reduction is calculated as follows:

| Waste reduction (%) = (*M*_CNC_ - *M*_WAAM_) / *M*_CNC_ × 100% | (1) |
| --- | --- |

where *M*_CNC_ and *M*_WAAM_ are the material consumption of the CNC and WAAM, respectively.

The material consumption *M*_WAAM_ in the “Weaving Love” project is 2400 kg using the WAAM technique. When choosing the CNC technique, its material consumption is calculated as:

| The cross-section area of 30mm hollow tube with a wall thickness of 4mm: *A*_tube_ = π × (15^2^ - 11^2^) = 326.7 mm^2^ | (2) |
| --- | --- |
| Corresponding solid block area: *A*_block_ = π × (15^2^) = 706.9 mm^2^ | (3) |
| Material multiplier = 706.9 / 326.7 = 2.16 | (4) |

The raw material requirement using the CNC considering at least 60% waste is:

| *M*_CNC_ = 2400 kg × 2.16 / (1-0.6) = 12,960 kg ≈ 13,000 kg | (5) |
| --- | --- |

The waste reduction is:

| Waste reduction (%) = (13,000 – 2,400) / 13,000 × 100% = 81.5% | (6) |
| --- | --- |

## ***Energy Saving***

The expression of energy saving is calculated as follows:

| Energy saving (%) = (*E*_CNC_ - *E*_WAAM_) / *E*_CNC_ × 100% | (7) |
| --- | --- |

where *E*_CNC_ and *E*_WAAM_ are the energy saving of the CNC and WAAM, respectively. The energy intensity is 50 kWh/kg [41].

The *E*_WAAM_ consumed in the “Weaving Love” project is calculated as:

| *E*_WAAM_ = 2,400 kg × 50 kWh/kg = 120,000 kWh | (8) |
| --- | --- |

The *E*_CNC_ is calculated as:

| *E*_CNC_ = 13,000 kg × 50 kWh/kg = 650,000 kWh | (9) |
| --- | --- |

The energy saving is:

| Energy saving (%) = (650,000 – 120,000) / 650,000 × 100% = 81.5% | (10) |
| --- | --- |

## ***Manufacturing Time Reduction***

The expression of time saving is calculated as follows:

| Time saving (%) = (*T*_CNC_ - *T*_WAAM_) / *T*_CNC_ × 100% | (11) |
| --- | --- |

where *T*_CNC_ and *T*_WAAM_ are the time consumption when using CNC and WAAM in manufacturing, respectively.

The *T*_WAAM_ is calculated with the deposition rate 0.95 kg/hour, given as:

| *T*_WAAM_ = 2,400 kg / 0.95 kg/hour = 2,526 hours ≈ 316 days ≈ 10.5 months (8 hours/workdays) | (12) |
| --- | --- |

The *T*_CNC_ is calculated with the machining rate 2 kg/hour, given as:

| *T*_CNC_ = 10,600 kg / 2 kg/hour = 5,300 hours ≈ 662.5 days ≈ 22.1 months (8 hours/workdays) | (13) |
| --- | --- |

The manufacturing time saving is:

| Time saving (%) = (22.1 – 10.5) / 22.1 × 100% = 52.5% | (14) |
| --- | --- |

## ***Operating Cost Saving***

The expression of operating cost saving is calculated as follows:

| Cost saving (%) = (*C*_CNC_ - *C*_WAAM_) / *C*_CNC_ × 100% | (15) |
| --- | --- |

The WAAM material cost of ER308L stainless steel weld wire: 50 HK$/kg [42], and the CNC material cost is 30 HK$/kg [43]. The labour and energy costs are 1200 HK$/day and 1.2 HK$/kWh, respectively.

The cost *C*_WAAM_ is calculated as:

| 2,400 kg × 50 HK$/kg + 316 days × 1,200 HK$/kg + 120,000 kWh ×1.2 HK$/kWh = 643,200 HK$ | (16) |
| --- | --- |

The cost *C*_CNC_ is calculated as:

| 13,000 kg × 30 HK$/kg + 662.5 days × 1,200 HK$/kg + 650,000 kWh ×1.2 HK$/kWh = 1,965,000 HK$ | (17) |
| --- | --- |

The operating cost saving is:

| Operating cost saving (%) = (1,965,000 – 643,200) / 1,965,000 × 100% = 67.3% | (18) |
| --- | --- |

## ***Embodied Carbon Reduction***

The expression of carbon reduction is:

| Carbon reduction (%) = (*CR*_CNC_ - *CR*_WAAM_) / *CR*_CNC_ × 100% | (19) |
| --- | --- |

The emission of CO_2_ per kilogram, namely the carbon factor is 6.15 kg CO_2_/kg [44].

The cost reduction of WAAM *CR*_WAAM_ is calculated as:

| 2,400 kg × 6.15 kg CO_2_/kg = 14,760 kg CO_2_ | (20) |
| --- | --- |

The cost reduction of CNC *CR*_CNC_ is calculated as:

| 13,000 kg × 6.15 kg CO_2_/kg = 79,950 kg CO_2_ | (21) |
| --- | --- |

The carbon reduction is:

| Carbon reduction (%) = (79,950 – 14,760) / 79,950 × 100% = 81.5% | (22) |
| --- | --- |

# **References**

[41] Kroll, L., et al., Lightweight components for energy-efficient machine tools. CIRP J. Manuf. Sci. Technol. **4(2)**, 148-160 (2011).

[42] Stainless Steel TIG/MIG AWS A5.9 Welding Wire ER308L. Available from: https://www.alibaba.com/product-detail/Stainless-Steel-TIG-MIG-WSA5_62389024933.html?spm=a2700.galleryofferlist.p_offer.d_image.276913a0IJd7vk&s=p.

[43] Asian Stainless Steel Prices. Available from: https://mepsinternational.com/gb/en/products/asia-stainless-steel-prices.

[44] Ecoinvent database for stainless steel.; Available from: https://ecoinvent.org/.
